# Inhibiting interferon-γ induced cancer intrinsic TNFRSF14 elevation restrains the malignant progression of glioblastoma

**DOI:** 10.1186/s13046-024-03131-7

**Published:** 2024-07-31

**Authors:** Yunhe Han, Cunyi Zou, Tianqi Liu, Wen Cheng, Peng Cheng, Anhua Wu

**Affiliations:** 1 Department of Neurosurgery, Shengjing Hospital of China Medical University, Shenyang, Liaoning 110004 China; 2 Department of Neurosurgery, The First Hospital of China Medical University, Shenyang, Liaoning 110001 China; 3 Institute of Health Sciences, China Medical University, Shenyang, Liaoning 110122 China

**Keywords:** Glioblastoma, TNFRSF14, Tumor microenvironment, Interferon, Immunotherapy

## Abstract

**Background:**

Prolonged interferon-γ signaling activation induces cancer resistance to therapeutics, especially immunotherapy. However, the detailed mechanisms are not well characterized. In present study, we explored cancer intrinsic resistant mechanisms employing for evading immune checkpoint blockade (ICB) and searched for key immune checkpoints contributing to the constitution of suppressive immune microenvironment of glioblastoma (GBM).

**Methods:**

We screened key immune checkpoint (IC) associated with IFN signaling activation in GBM according to integrated transcriptomic profiling on the ICs. Expression analysis and functional assays revealed that malignant cells elevated the key IC, TNFRSF14 expression under IFN-γ stimulation, which enhanced their proliferation and in vivo tumorigenicity. Therapeutic efficiency of TNFRSF14 disruption in GBM was evaluated with in vitro and in vivo functional assays, including immunofluorescence, transwell, RT-qPCR, flow cytometry, mass cytometry, and mice preclinical GBM models. Moreover, the improvement of TNFRSF14 blockade on the efficacy of PD-L1 treatment was examined in mice intracranial xenograft bearing models.

**Results:**

TNFRSF14, a previously poorly characterized IC, was disclosed as a checkpoint with malignant intrinsic elevation closely associated with type II not type I IFN signaling activation in GBM. Anti-PD-L1 treatment induces compensatory TNFRSF14 elevation, while enhancing IFN-γ production. TNFRSF14 phosphorylates FAK at Y397 and consequently activates NF-κB, which not only strengthens the tumorigenicity of GBM cells, but also enhances TAMs recruitment through elevating CXCL1/CXCL5 secretion from GBM cells. TNFRSF14 ablation reduces the tumorigenicity of GBM cells, reshapes the immunosuppressive microenvironment, and enhances therapeutic efficacy of anti-PD-L1 in mouse orthotopic GBM model.

**Conclusion:**

Our findings highlight a malignant TNFRSF14/FAK axis as a potential target to blunt cancer-intrinsic resistance to ICB treatment, which may help improve the therapeutic efficiency of immunotherapy in malignancies.

**Supplementary Information:**

The online version contains supplementary material available at 10.1186/s13046-024-03131-7.

## Background

During the past decade, immune checkpoint blockade (ICB) is the most encouraging advancement in cancer therapy and has achieved success in multiple types of malignant tumors [1]. However, primary or acquired resistance to ICB is very common in cancer patients, which mechanisms remain to be characterized. Previous reports disclose that interferon (IFN) signaling activation plays a vital role in anti-tumor immune response in currently widely used ICB treatments [2–4]. Nevertheless, type I and type II IFN signaling and sensing pathways could be hijacked by malignant cells to facilitate their survival and escape immunosurveillance [1, 5, 6], which eliminates effective response to ICB and even induces cancer hyper-progression during immunotherapy [7]. There may be other compensatory immune checkpoints (ICs) to facilitate this functional shift of IFN response during ICB treatment, which is necessary to be identified.

As the most common primary malignant tumor in adult brain, glioblastoma (GBM) is notorious for its aggressiveness and dismal prognosis. It has been implicated that IFN signaling is significantly activated in GBM and closely correlated to the progression and unfavorable survival in GBM [8]. Tumor associated macrophages (TAMs) and malignant cell undergoing immunogenic cell death (ICD) may serve as the source of producing IFN-γ in its tumor microenvironment (TME), which leads to downstream signaling activation [9, 10]. IFN-γ signaling activation has been proposed to induce transcriptomic mesenchymal signature changes in GBM cells, which contributes to the constitution of immunosuppressive TME [9]. These observations support the multifaceted roles of IFN-γ signaling activation in this devastating tumor. Upregulation of alternative inhibitory IC is one of pivotal ways for malignant cells mediating IFN-γ-driven resistance to ICB among various mechanisms in cancer [6, 11]. However, the detailed mechanisms are not well understood.

Here, according to integrated transcriptomic profiling on the ICs associated with type I and type II IFN signaling activation in GBM, we identify TNFRSF14 (TNF receptor superfamily member 14, also named as HVEM) as a key IC associated with IFN signaling activation in GBM. Expression analysis and functional assays reveals that GBM cells elevate their TNFRSF14 expression under IFN-γ (type II IFN signaling), instead of IFN-α or IFN-β (type I IFN signaling) stimulation, which enhances their proliferation and in vivo tumorigenicity. Mechanistically, TNFRSF14 upregulation in GBM cells augments FAK phosphorylation at Y397, which boosts GBM cell malignant behaviors through activating NF-κB. Moreover, GBM intrinsic TNFRSF14 upregulation promotes the recruitment of anti-inflammatory TAMs by enhancing CXCL1/CXCL5 secretion from GBM cells. TNFRSF14 blockade improves the efficiency of anti-PD-L1 therapy and extends the survival of mice bearing intracranial xenograft, highlighting the clinical translation potential of this immune checkpoint.

## Materials and methods

### Mice experiments

After anesthesia with isoflurane, C57BL/6 (male, 6–8 weeks) or BALB/c Nude Mice (male, 6–8 weeks) were fixed with mouse stereotactic devices, and then 3 µl GBM cell suspension was injected into the mouse brains at a depth of 3.0 mm [12]. Intracranial orthotopic mouse model transplanted with GL261 cells and mGSCs was employed for in vivo anti-TNFRSF14 and anti-PD-L1 blockade experiments, and mouse PD-L1 (10 µg/g body weight, BE0101, InVivoMab, BioXcell) or TNFRSF14 antibody (LH1, Functional Grade, 5 µg/g body weight, ebioscience, 16-5962-38) were injected intraperitoneally at the 5th, 8th, 11th day after intracranial cells transplantation.

### Cell lines

Primary patient-derived glioma sphere cells (GSC1) were derived from fresh GBM resection sample by methods described before [13]. Briefly, after tumor resection, the tumor samples were dissociated to single cells with mechanical and enzymatic methods (acctuase), and then were recovered and cultured in stem cell medium (DMEM/F-12 medium supplemented with B27 supplement, 20 ng/ml EGF, and 20 ng/ml FGF). Mouse glioma stem cells (mGSCs) were collected from spontaneous GBM models established by sleeping beauty (SB) transposon method as previously described [14]. The primary glioma sphere cell line was cultured less than 20 passages and maintained in DMEM/F-12 medium (10,565,018, Gibco) containing 1X B27 supplement (17,504,044, Gibco), epidermal growth factor (EGF, 20 ng/ml, AF-100-15, Peprotech), basic fibroblast growth factor (bFGF, 20 ng/ml, AF-100-18B, Peprotech) and heparin (2.5 µg/ml, H3149, Sigma). U87 and GL261 cell lines were purchased from the Chinese Academy of Sciences cell bank (Shanghai, China), and American Type Culture Collection (ATCC, Manassas, VA, USA), respectively. U87 and GL261 cells were maintained in Dulbecco’s Modified Eagle Medium (DMEM) containing 10% fetal bovine serum (FBS) and 1% penicillin/streptomycin at 37 °C with 5% CO_2_. THP-1 cells were obtained from National Collection of Authenticated Cell Cultures (NCACC, Shanghai, China). THP-1 cells were maintained in RPMI-1640 medium, supplemented with 10% fetal bovine serum (FBS) and 1% penicillin/streptomycin (Gibco) at 37 °C with 5% CO_2_. THP-1 cells were primed with 5 nM PMA (Sigma) for 48 h to obtain THP1-derived macrophages. Mouse bone marrow-derived macrophages (BMDMs) were harvested from six-week-old C57BL/6 male mice and cultured in RPMI-1640 supplemented with 10% FBS and 20 ng/mL M-CSF (Peprotech, Cat#315-02, NJ, USA) for 7 days with medium replenished every 3 days as previously described [15].

### Immunohistochemistry (IHC) and IHC scoring

A streptavidin-biotin immunostaining method was employed and evaluated as described before [12]. The immunohistochemistry staining and survival analysis of TNFRSF14 in CMU samples were described as before [8]. Briefly, tissue sections, including human and mouse samples, were fixed in 4% paraformaldehyde, embedded in paraffin, and cut into 4 μm sections. All slides were dewaxed with xylene/ethanol, and then antigen retrieval was performed in a microwave oven. The sections were reacted with primary antibodies after blocking. Diaminobenzidine tetrahydroxy chloride (DAB) solution was used to visualize the peroxidase activity. Then sections were stained with hematoxylin. The IHC staining intensities were evaluated with German immunohistochemical score (GIS) [8, 9].

### Immunofluorescence

An immunofluorescence method was employed and evaluated as described before [12]. Briefly, 3 µm thick section slides from frozen human tissue were washed three times in PBS. Then the sections were permeabilized with 0.5% Triton X-100 (T8200, Solarbio) for 15 min. After 3% BSA incubation for 1.5 h, sections were incubated in primary antibodies at 4°C overnight. Following incubation with fluorescein (FITC) or rhodamine (TRITC) secondary antibody and 4’,6-diamidino-2-phenylindole (DAPI, C0060, Solarbio), the samples were detected using a confocal microscope (FV1000S-SIM; Olympus). The images were merged digitally to monitor the co-localization condition.

### Proteome profiler human array

Human proteome profiler array kits for chemokines (#ARY017, R&D Inc.) and phosphor-kinases (#ARY003C, R&D Inc.) were employed to screen cytokines and phospho-kinases associated with TNFRSF14, following the manufacturer’s protocol. The relative densities of specific protein expression were determined using ImageJ software. The detailed information was provided in Table S1.

### Small interfering RNA (siRNA) and lentivirus mediated gene knockdown and overexpression

siRNAs were obtained from Sangon Biotech (Shanghai, China). Lipofectamine 3000 (Life Technologies) was employed for siRNAs transfection, according to the manufacturer’s instructions. Lentiviral TNFRSF14 knockdown and overexpression vectors were obtained from Gene-Chem (Shanghai, China). After transduction, the cells were screened with 10 µg/ml puromycin for 15 days. The detailed sequences of siRNA shRNA employed in present study were listed in Table S2.

### RNA isolation and reverse-transcription quantitative PCR (RT-qPCR)

Total RNA was isolated using TRIzol (TaKaRa), and cDNA was synthesized using the Prime-Script RT Master Mix (TaKaRa). Quantitative PCR (PCR LightCycler480, Roche) was detected with SYBR Green Master Mix (TaKaRa). Each sample was run in three replicates.18s was used as the control in human cell lines and Gapdh was used as control in mouse cell line. The PCR primers were listed in Table S3.

### In vitro cell proliferation assays

Cell proliferation was measured with MTS using a Cell Titer 96^®^ AQueous Non-Radioactive cell proliferation assay kit (Promega) according to the manufacturer’s instructions. Cells were cultured in 96-well plates at a density of 1 × 10^3^ cells/well for 24, 48, 72, 96 and 120 h. Then, 20 µl of MTS were added into each well, followed by 3 h incubation at 37 °C. The microplate reader (VICTOR Nivo, PerkinElmer) was used to detect the absorbance at 490 nm.

### Cell migration and invasion assays

Transwell chambers with 8 μm pores (Corning) were employed for cell migration and invasion assays (coating with 50 µl Matrigel, BD Biosciences). Cells were allowed to invade the filters toward the lower compartment for 20 h. Crystal violet was applied for the staining of invasive and migrating cells, then counted, and photographed using a microscope (DM 2500 LED, LEICA, USA).

### Co-immunoprecipitation (Co-IP)

Total proteins were extracted with whole cell lysis buffer (Beyotime Biotechnology, Beijing, China). After centrifuge. anti-TNFRSF14 antibody (ab62462, Abcam, 1 µg) or anti-FAK antibody (#71,433, Cellsignalingtech,1ug).

were added into 200 µl cell lysates. After incubation at 4 °C overnight, mix protein A/G magnetic beads (MedChem Express) were added into the cell lysates and followed by 2 h rotating incubation for and then centrifuged. The precipitates were washed 5 times with wash buffer and followed by immunoblotting.

### Enzyme linked immunosorbent assay (ELISA)

The culture medium was harvested and centrifuged for 20 min at 4 °C,1000 g. Then, the supernatant was collected and examined with ELISA kits according to manufacturer’s protocol (CXCL1 and CXCL5, SEA041Hu, SEA860Hu, Cloud-clone). The microplate reader (PerkinElmer VICTOR Nivo) was applied for conducting the measurement.

### Flow cytometry

Flow cytometry was performed as previously described [13]. Briefly, single cell suspension was incubated at 4 °C for 30 min with antibody listed in Table S1. The IFN-γ and TNF-α intracellular staining were performed with Flow Cytometry Fixation & Permeabilization Buffer Kit I (#FC009, R&D). Matched non-specific isotype immunoglobulins were served as controls. Cells were detected by BD LSR Fortessa flow cytometer (BD Biosciences), and the results were analyzed by FlowJo V10 software (TreeStar).

### Cytometry by time-of-flight mass cytometry (CyTOF)

CyTOF analysis was performed as previously described [12]. Briefly, tumor tissue was digested into single-cell suspension by DNase and 0.25% trypsin. Then, a metal-labeled antibody cocktail was applied to map tumor immune microenvironment. T-distributed stochastic neighbor embedding (tSNE), followed by K-Nearest Neighbor (KNN) clustering, was used to distinguish specific immune cell populations.

### Establishing IFN signaling activation score

The gene lists of type I and type II IFN signaling were obtained from MsigDB signatures HALLMARK INTERFERON ALPHA RESPONSE and HALLMARK INTERFERON GAMMA RESPONSE. The type I and type II IFN signaling score for each sample was calculated with the average expression of the genes belonging to the corresponding term as previously described [16, 17]. Pearson correlation analysis was used to analyze the correlation between immune checkpoints and IFN signaling score.

### Single-cell transcriptomic analysis

Seurat V4.0.0 (RRID: SCR_016341) was employed for the normalization and clustering of single-cell RNA-sequencing dataset (scRNA-seq, GSE131928). Batch-corrected and data integration were used LIGER (Linked Inference of Genomic Experimental Relationships). Then, the gene expression counts were normalized to the library size and log2-transformed. Principal component analyses were used to reduce the dimensionality by using the top 5000 most variable genes.

### Statistical analysis

Statistical analyses were conducted with Prism 7 and R 3.4. A two-tailed t-test or one-way ANOVA was used for distinguishing the differences between or among the groups, and *p* < 0.05 was considered statistically significant. Correlation analysis was performed with Pearson method. Survival distribution was estimated using Kaplan-Meier analysis, and log-rank test was applied to evaluate differences between stratified groups. All experiments were performed at less three replicates unless mentioned elsewhere.

## Results

### TNFRSF14 is an IC closely relevant to IFN signaling activation in GBM

**Fig. 1:**
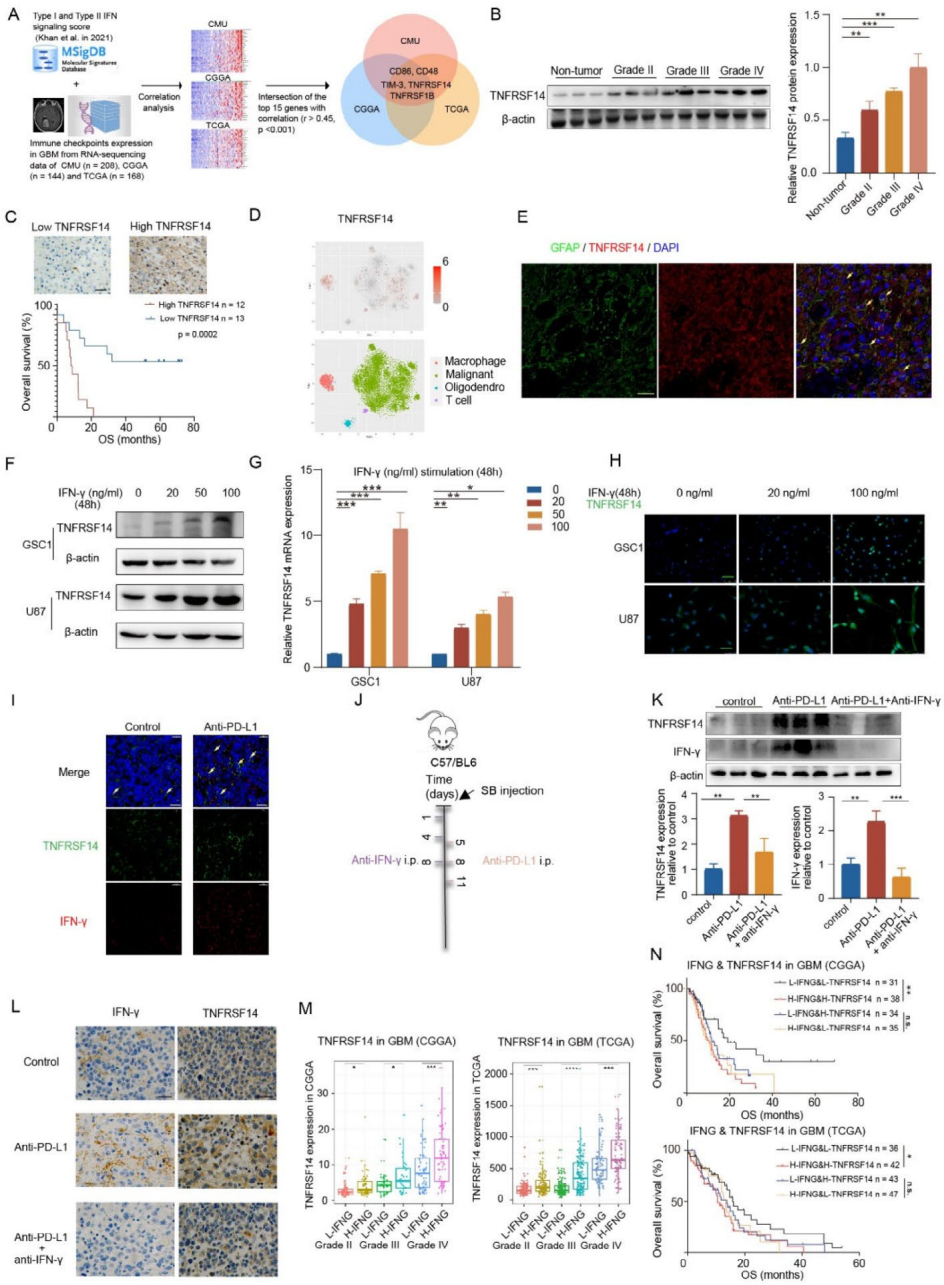
TNFRSF14 is an immune checkpoint elevated with the activation of IFN signaling in GBM. (**A**) Intersection of the overlapping immune checkpoints significantly relevant to IFN signaling in GBM, according to CMU, CGGA and TCGA cohorts (CMU *n* = 208, CGGA *n* = 144, TCGA *n* = 168, Pearson correlation analysis, specific r and p value were showed in Table S6). (**B**) Representative western blotting images and analyses of TNFRSF14 in CMU glioma samples (relative to Grade IV, *n* = 3 for each grade, one-way ANOVA). (**C**) Representative immunohistochemical staining images (upper) and survival analysis (lower, log-rank, *n* = 25) of TNFRSF14 in GBM samples (scale bar, 25 μm). (**D**) Analysis of TNFRSF14 expression in single-cell GBM RNA-seq dataset (lower: cell subpopulation distribution map; upper: result of single cell analysis) (GSE131928). (**E**) Representative immunofluorescence images of TNFRSF14 and GFAP in GBM samples (scale bar, 25 μm). (**F**) Representative western blotting images of indicated GBM cells with IFN-γ stimulation (0, 20, 50, and 100 ng/ml; 48 h). (**G**) qPCR analyses of TNFRSF14 in GBM cells with indicated IFN-γ stimulation (relative to 0 ng/ml, *n* = 3 for each concentration, one-way ANOVA). (**H**) Representative immunofluorescence images of TNFRSF14 in GBM cells (GSC1 and U87) with indicated IFN-γ stimulation (0, 20, 100 ng/ml; scale bar, 75 μm). (**I**) Representative immunofluorescence images of TNFRSF14 (green) and IFN-γ (red) in mice GBM with anti-PD-L1 treatment (scale bar, 25 μm). (**J**) Schematic graph of anti-PD-L1 and anti-IFN-γ treatment in indicated GBM mice model (SB: mGSCs derived from mice GBM established by sleeping beauty transposon vector). (**K**) Representative western blotting images and analyses of TNFRSF14 and IFN-γ of tumor tissue from indicated GBM mice model. (**L**) Representative immunohistochemical staining image of IFN-γ and TNFRSF14 in brain sections from indicated GBM mice model (scale bar, 25 μm). (**M**) Transcriptomic analysis showed TNFRSF14 high-expression was significantly associated with IFN-γ elevation in GBM (CGGA: grade II low *n* = 52, high *n* = 53, grade III low *n* = 33, high *n* = 34, grade IV low *n* = 69, high *n* = 69; TCGA: grade II low *n* = 111, high *n* = 112, grade III low *n* = 122. high *n* = 123, grade IV low *n* = 84. high *n* = 84, t-test). (**N**) The co-elevation of IFN-γ and TNFRSF14 was associated with unfavorable patient survival in GBM (CGGA: low-low *n* = 31, high-high *n* = 38, low-high *n* = 34, high-low *n* = 35; TCGA: low-low *n* = 36, high-high *n* = 42, low-high *n* = 43, high-low *n* = 47, log-rank). (n.s. *p* ≥ 0.05, * *p* < 0.05, ** *p* < 0.01, *** *p* < 0.001, **** *p* < 0.0001)

To screen the ICs associated with IFN signaling activation in GBM, we firstly summarized a list of 67 ICs encoding genes (Table S4). According to HALLMARK INTERFERON ALPHA RESPONSE and HALLMARK INTERFERON GAMMA RESPONSE gene sets in MsigDB [16], type I and type II IFN signaling activation score was calculated [17]. Then, we performed Pearson correlation analysis between 67 IC genes and IFN signaling activation score (type I and type II, respectively) among RNA sequencing (RNA-seq) data from clinical GBM samples (CMU cohort, *n* = 208) and two public independent GBM transcriptomic datasets (The Cancer Genome Atlas, TCGA *n* = 168, and Chinese Glioma Genome Atlas, CGGA *n* = 144) (Table S5). After intersecting the top 15 ICs which expression was most positively associated with IFN type I and type II score in these three GBM RNA-seq datasets, CD86, CD48, TIM-3, TNFRSF14 and TNFRSF1B were disclosed as five overlapping genes strongly related to IFN signaling (Fig. 1A, Figure S1A-C and Table S6). Then, survival analysis disclosed that TNFRSF14 was the only immune bidirectional (co-inhibitory and co-stimulatory) checkpoint associated with poor prognosis in GBM. Therefore, we selected TNFRSF14 as the candidate for further investigation. Western blotting analysis with clinical samples disclosed that TNFRSF14 expression elevated with increased glioma grades (Fig. 1B), which is consistent with a previous report [18]. IHC analysis further revealed that GBM patients with TNFRSF14 upregulation exhibited a more unfavorable survival compared to patients with low TNFRSF14 expression (Fig. 1C). Similar data was obtained from TCGA and CGGA databases (Figure S1D).

Next, we sought to delineate the cell populations expressing TNFRSF14. TNFRSF14 was originally reported as an immune checkpoint expressed by immune cells including macrophages and T cells [19]. Interestingly, with interrogating scRNA-seq data from 28 GBM patients (GSE131928) [20], we found that malignant cells and TAMs were two major cell populations with TNFRSF14 expression in GBM (Fig. 1D). Immunofluorescence analysis in clinical GBM samples demonstrated that there were malignant cells with co-staining of TNFRSF14 and GFAP, indicating cancer intrinsic TNFRSF14 expression in GBM (Fig. 1E). Additionally, ligands of TNFRSF14 were barely detected in the above single cell GBM RNA-seq datasets (Figure S1E). Given the driving role of malignant cells in cancer initiation and progression and the current limited understanding on cancer intrinsic IC mechanisms, we selected the functions of cancer cell intrinsic TNFRSF14 for further investigation.

It has been reported that IFN-γ serving as a central node in establishing and maintaining immune equilibrium instead of IFN-α and IFN-β [21]. Then, we employed IFN-α, IFN-β, and IFN-γ (0, 20, 50, 100 ng/ml) to incubate two GBM cell lines (one primary GBM cell line, GSC1, and one conventional GBM cell line, U87) to induce IFN signaling activation in GBM cells in vitro. Western blotting analysis disclosed that after incubation, the expression of TNFRSF14 in GBM cells was up-regulated only after IFN-γ treatment, instead of IFN-α and IFN-β (Fig. 1F and Figure S1I). Furthermore, PCR analysis disclosed that IFN-γ stimulation did induce a dose-dependent TNFRSF14 elevation in GBM cells (Fig. 1G), which supports a close association of TNFRSF14 elevation with IFN-γ signaling activation in GBM cells. Immunofluorescence staining analysis also revealed that the expression of TNFRSF14 in GBM cells was up-regulated after IFN-γ treatment (Fig. 1H). Previously, to explore the efficacy of anti-PD-L1 treatment in GBM, we employed a sleeping beauty (SB) transposon orthotopic xenograft-bearing murine model treated with anti-PD-L1 therapy, which revealed that anti-PD-L1 monotherapy had no significant inhibition on GBM growth [12]. With examining by immunofluorescence staining in these samples, we found that PD-L1 blockade induced IFN-γ upregulation in these tumors, as well as TNFRSF14 elevation (Fig. 1I). Since anti-PD-L1 could induce IFN-γ elevation (Fig. 1I) but had no significant effect on survival, we hypothesized that there might be underlying mechanisms mediating the tolerance to anti-PD-L1 treatment, which restrained the efficacy of anti-PD-L1 in GBM. This indicates that GBM cells may upregulate TNFRSF14 expression as a potential mechanism of IFN-γ-driven tolerance to anti-PD-L1 treatment. This was further supported by the data obtained from mice preclinical models. Neutralizing upregulated IFN-γ induced by PD-L1 treatment significantly attenuated TNFRSF14 expression in tumor derived from mGSCs in immune competent mice model (Fig. 1J-L and Figure S1F). Meanwhile, transcriptomic analysis of clinical glioma samples showed that a tendency of IFN-γ upregulation accompanied by TNFRSF14 elevation in both LGG and GBM (Fig. 1M and Figure S1G). Additionally, enhanced IFN-γ expression accompanied with TNFRSF14 elevation (High-IFNG & High-TNFRSF14) indicated a shorter survival in glioma than the lower counterpart (Low-IFNG & Low-TNFRSF14), while similar results couldn’t be obtained from PD-L1 (Fig. 1N and Figure S1H). Together, these results indicate that there is a cancer intrinsic TNFRSF14 elevation in GBM accompanying with IFN-γ signaling activation, which may be a potential compensatory mechanism to IFN-γ exposure in GBM and indicate poor prognosis of GBM patients.

### GBM intrinsic TNFRSF14 elevation augments GBM cell tumorigenicity

**Fig. 2:**
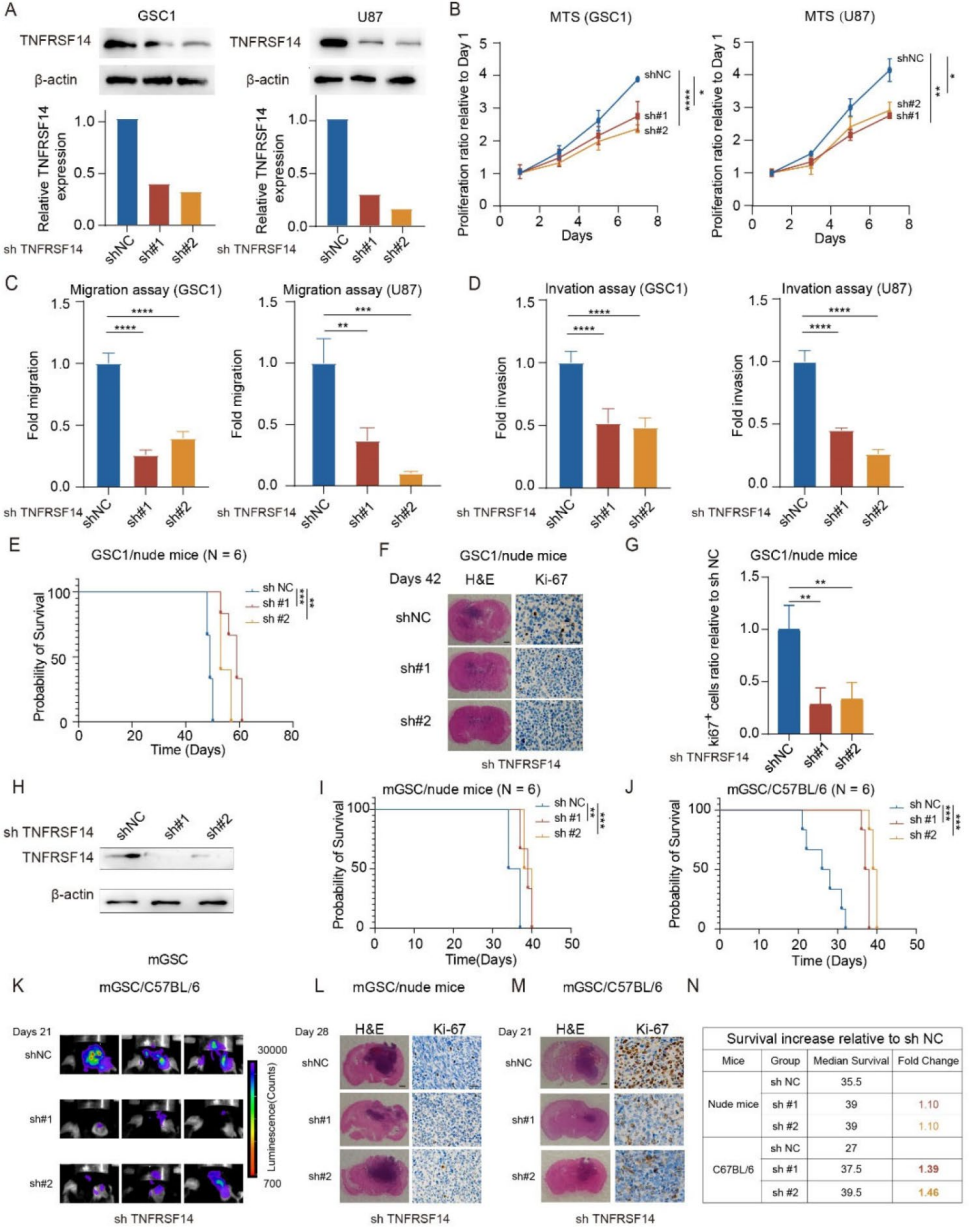
Disrupting malignant intrinsic TNFRSF14 renders GBM growth. (**A**) Representative western blotting images and analysis of TNFRSF14 in indicated GBM cells transduced with shTNRSF14 or non-targeting vector (relative to shNC). (**B**) MTS assay showing TNFRSF14 knockdown significantly reduced the growth of indicated GBM cells (*n* = 3, one-way ANOVA). (**C**, **D**) In vitro migration (**C**) and invasion assays (**D**) of indicated GBM cell migration (relative to shNC, *n* = 3, one-way ANOVA). (**E**) Survival analysis of intracranial orthotopic in nude mice with indicated GSC1 (*n* = 6, log-rank). (**F**) H&E and Ki-67 staining images of indicated mice brain section (left scale bar, 500 μm; right scale bar, 25 μm). (**G**) Analysis of Ki-67 immunohistochemical staining in indicated mice brain sections (*n* = 3, one-way ANOVA). (**H**) Representative western blotting images of TNFRSF14 in indicated mGSC samples (relative to shNC). (**I**, **J**) Survival analysis of mice intracranially transplanted with indicated mGSCs in nude mice (**I**) and C57BL/6 mice (**J**) (*n* = 6, log-rank). (**K**) Bioluminescence images of mice brains transplanted with indicated mGSCs in C57BL/6 mice. (**L**, **M**) H&E and Ki-67 staining images of indicated mice brain section (scale bar: left 500 μm, right 25 μm). (**N**) The comparison of fold change of median survival extended by TNFRSF14 knockdown between intracranial mGSC transplantation in immunodeficient (Nude mice) and immune component mice model (C57BL/6). (* *p* < 0.05, ** *p* < 0.01, *** *p* < 0.001, **** *p* < 0.0001)

To reveal whether intrinsic TNFRSF14 elevation in GBM cells impacts their proliferation and tumorigenicity, we performed TNFRSF14 knockdown in GSC1 and U87 cells by lentiviral vectors, respectively. After TNFRSF14 knockdown (Fig. 2A), the proliferation capabilities of these GBM cells were significantly reduced (Fig. 2B), as well as their migration and invasion abilities (Fig. 2C, D and Figure S2A, B). Overexpressed TNFRSF14 promoted the proliferation capabilities of tumor cells (Figure S2C and S2D). To confirm whether GBM intrinsic TNFRSF14 affected tumorigenicity of GBM cells in vivo, we applied mouse orthotopic xenograft model and intracranially transplanted GSC1 cells into immune-deficient nude mice. We observed not only an extended survival in TNFRSF14 knock-down group mice (Fig. 2E), but also remarkably reduced tumor volume and decreased Ki-67 staining intensity in tumor samples from these mice (Fig. 2F and G). Similar results were obtained from C57BL/6 mice orthotopic transplanted with GL261 cells (Figure S2E-K). To further confirm the role of cancer intrinsic TNFRSF14 in promoting the tumorigenicity of GBM cells, mGSCs isolated from mouse sleeping beauty transposon derived GBM [13] were transduced with lentiviral shTNFRSF14 or control vector, respectively. Then, mGSCs were orthotopic transplanted into immunocompetent C57BL/6 mice or immunodeficient BALB/c nude mice, respectively. Tnfrsf14 knockdown in mGSCs (Fig. 2H) significantly restrained their tumorigenicity and extended the survival of tumor-bearing mice (Fig. 2I and J), with reduced tumor volume and lower Ki-67 expression (Fig. 2K-M). Notably, the average survival extended by Tnfrsf14 -knockdown mGSCs in immunocompetent mice was more significant than that in immunodeficient mice (Fig. 2I, J and N). This implicates the potential survival benefit brought by TNFRSF14 blockade through restraining pro-tumorigenic effect of non-tumor cell components in TME. Collectively, these data suggest a promoting role of cancer intrinsic TNFRSF14 on the tumorgenicity of GBM cells.

### TNFRSF14 elevation enhances the recruitment of anti-inflammatory TAMs

**Fig. 3:**
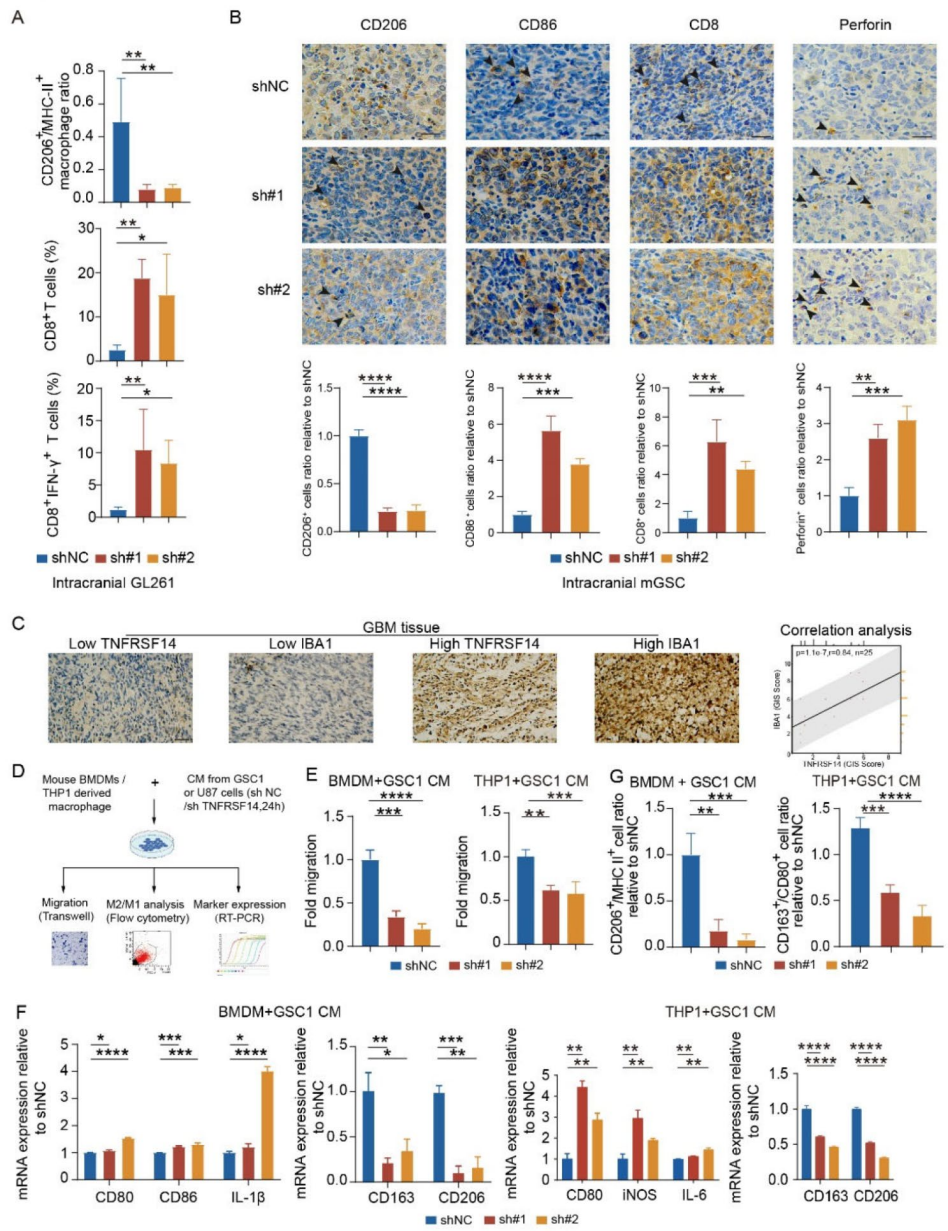
Glioma cell TNFRSF14 enhances anti-inflammatory TAM recruitment. (**A**) Flow cytometry analysis of indicated markers (CD206^+^, MHC II^+^, CD8^+^ or IFN-γ^+^) in tumor samples from mice intracranially transplanted with indicated GL261 cells (*n* = 3, one-way ANOVA). (**B**) Representative immunohistochemical staining images and analysis (*n* = 3, one-way ANOVA) of indicated markers (CD206, CD86, CD8, and perforin) in brain section of mice intracranially transplanted with indicated mGSCs (scale bar, 25 μm). (**C**) Representative immunohistochemical images and Pearson correlation analysis of TNFRSF14 and IBA1 in clinical GBM samples (*n* = 25, scale bar, 25 μm). (**D**) Schematic diagram of THP1- and BMDM-derived macrophages treated with conditioned medium of indicated GBM cells. (**E**) In vitro migration assays of THP1- and BMDM-derived macrophages treated with conditioned medium from indicated GBM cell (relative to shNC, *n* = 3, one-way ANOVA). (**F**) qPCR analyses of indicated markers in THP1- and BMDM-derived macrophages incubated with indicated conditioned medium treatment (relative to shNC, *n* = 3; one-way ANOVA). (**G**) Flow cytometry analysis of CD206^+^/MHC II^+^ in BMDM-derived macrophages (left) and CD163^+^/CD80^+^ in THP1-derived macrophages (right) with indicated conditioned medium treatment (*n* = 3, one-way ANOVA). (* *p* < 0.05, ** *p* < 0.01, *** *p* < 0.001, **** *p* < 0.0001)

Next, we sought to evaluate whether cancer intrinsic TNFRSF14 contributes to the constitution of immunosuppressive TME in GBM. We employed mouse immune competent orthotopic GBM cell transplantation model to characterize TME remodeled by cancer intrinsic TNFRSF14 in GBM. According to FACS analysis, TNFRSF14 ablation in mouse GBM cells efficiently decreased the ratio of CD206^+^/MHC II^+^ TAMs in mouse GBM tumor tissues. Among the infiltrating cells, more CD8^+^ T cells and CD8^+^ IFN-γ^+^ T cells were detected (Fig. 3A and Figure S3A-C). Moreover, IHC staining analysis demonstrated that more CD86^+^ TAMs, CD8^+^ T cells and perforin were observed in tumor samples derived from Tnfrsf14-knockdown GL261 (Figure S3E) and mGSC cells (Fig. 3B). This indicated that TNFRSF14 not only influenced malignant behaviors of GBM cells, but also contributed to remodeling TME via crosstalk with TAMs, which facilitated immune evasion of GBM cells. Indeed, IHC staining analysis revealed that TNFRSF14 expression in GBM patients was positively correlated with the expression of macrophage marker IBA1 (*p* < 0.0001, *r* = 0.84, Fig. 3C). We further employed human THP-1 cells and mouse BMDMs to perform in vitro functional assays to examine the potential influence of tumor-intrinsic TNFRSF14 on macrophages (Fig. 3D). Indeed, THP-1 and BMDMs-derived macrophages treated with conditioned medium (CM) from human or mouse GBM cells with TNFRSF14 knockdown showed a significantly decreased chemotaxis (Fig. 3E and Figure S3D, F) and anti-inflammatory polarization capabilities, compared to CM from control group (Fig. 3F, G and Figure S3F-J). qPCR analysis also revealed that mRNA of pro-inflammatory markers (CD80, iNOS and IL-6) significant increased while anti-inflammatory markers (CD163 and CD206) decreased in both of THP1- and BMDMs-derived macrophages with treatment of CM from TNFRSF14 knockdown human or mouse GBM cells (Fig. 3F and Figure S3G). Then, FACS analysis was applied to examine macrophage phenotypes. Consistently, THP1- and BMDMs-derived macrophages cocultured with CM from TNFRSF14 knockdown GBM cells showed a decreased ratio of CD163 and CD80 positive cell population compared to the samples treated with CM from control group (Fig. 3G and Figure S3H-J). Together, these results support that malignant cell intrinsic TNFRSF14 is involved in the recruitment of TAMs and promotes their polarization to anti-inflammatory phenotype in GBM.

### FAK serves as the downstream effector of TNFRSF14 in GBM cells and facilitated nuclear translocation of NF-κB

**Fig. 4:**
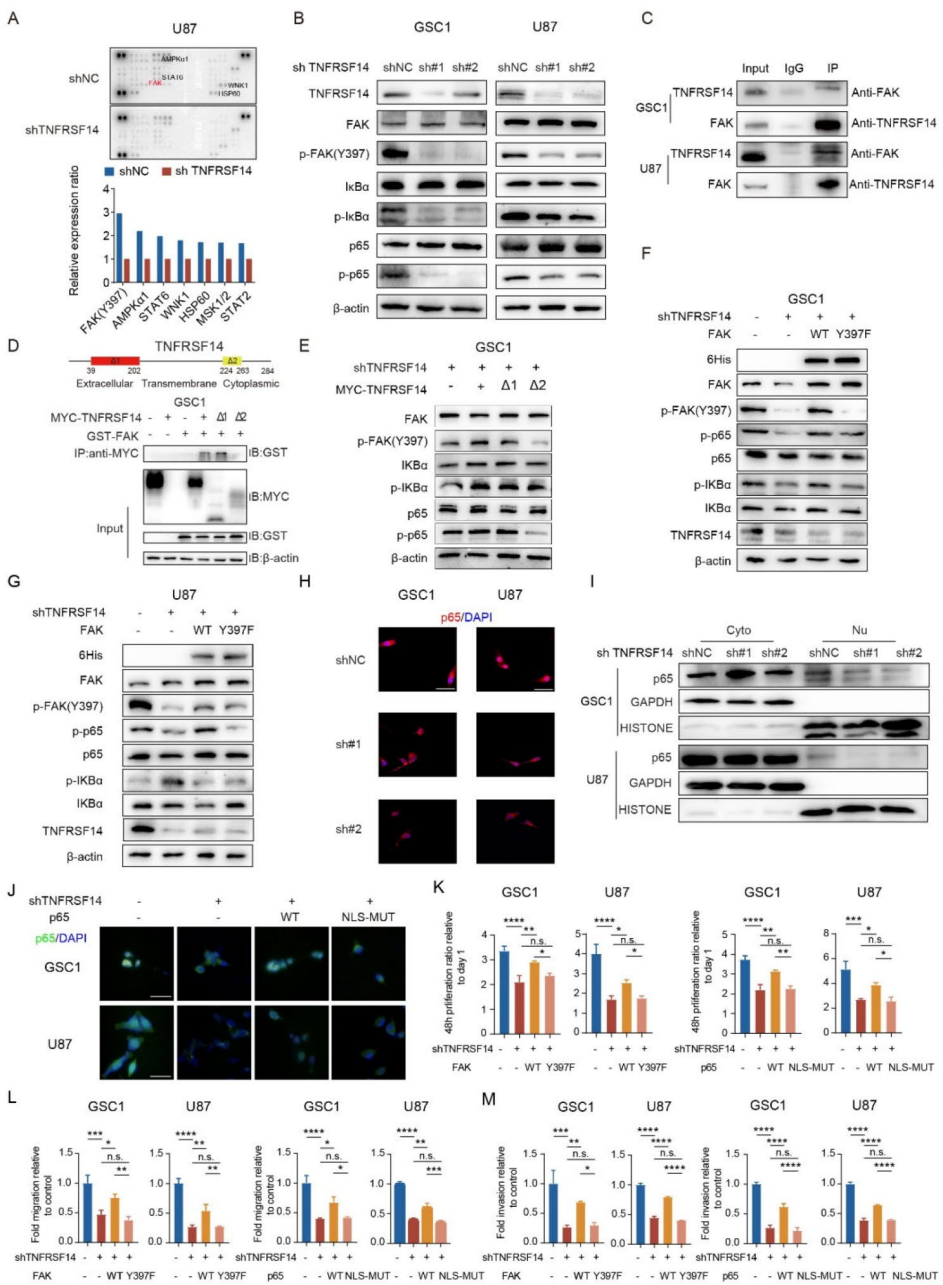
GBM cell TNFRSF14 functions through phosphorylating FAK at Y397 and promoting nuclear translocation of NF-κB. (**A**) Representative images (upper) and analyses (lower) of screening with human Phospho-Kinase Array showing FAK as a downstream effector of TNFRSF14 (relative to sh NC). (**B**) Representative western blotting images of indicated markers in GSC1 cells and U87 transduced with TNFRSF14 knockdown or non-targeting vectors, respectively. (**C**) Co-immunoprecipitation analysis of TNFRSF14 and FAK interaction in indicated GBM cells. (**D**) Co-immunoprecipitation analysis of interaction among FAK and different TNFRSF14 fragments in GSC1 cells. (**E**) Representative western blotting images of indicated markers in GSC1 cells treated with different TNFRSF14 fragment overexpression. (**F**) Representative western blotting images of indicated markers in GSC1 cells transfected with FAK wild type or Y397F mutant after TNFRSF14 knockdown. (**G**) Representative western blotting images of indicated markers in U87 cells transfected with FAK wild type or Y397F mutant after TNFRSF14 knockdown. (**H**) Immunofluorescence staining of p65 cytoplasmic localization in indicated GBM cells (scale bar, 75 μm). (**I**) Western blotting analysis of nucleus and cytoplasm p65 in indicated GBM cells. (**J**) Immunocytochemical staining of p65 cytoplasmic localization in indicated GBM cells transfected with p65 wild type or nuclear localization sequence mutant after TNFRSF14 knockdown (scale bar, 75 μm). (**K**-**M**) In vitro proliferation (**K**), migration (**L**) and invasion (**M**) assays of GSC1 and U87 cell transfected with FAK wild type or Y397F mutant, p65 wild type or nuclear localization sequence mutant after TNFRSF14 knockdown. (*n* = 3, one-way ANOVA). (n.s. *p* ≥ 0.05, * *p* < 0.05, ** *p* < 0.01, *** *p* < 0.001, **** *p* < 0.0001)

Next, we sought to clarify the downstream effector of cancer intrinsic TNFRSF14 in GBM cells. Due to the key role of kinase in the transmission and activation of intracellular signaling pathways [2, 22], we employed a Human Phospho-Kinase Array to screen the potential downstream kinase affected by TNFRSF14. FAK Y397 phosphorylation was the most significantly reduced effector in the array induced by TNFRSF14 knockdown (Fig. 4A). The validation of western blotting analysis showed that knockdown of TNFRSF14 remarkably decreased FAK Y397 phosphorylation in GBM cells (Fig. 4B). To further examine the role of FAK in TNFRSF14 signaling, we applied a specific FAK Y397 phosphorylation inhibitor, Defactinib, to investigate whether FAK Y397 phosphorylation is involved in TNFRSF14-mediated malignant behavior regulation in GBM cells [23]. The results showed that Defactinib strongly weakened the enhanced proliferation, invasion and migration of GBM cells induced by TNFRSF14 overexpression (Figure S4A-C). These data support a vital role of FAK in regulating TNFRSF14-mediating malignant behaviors of GBM cells. Moreover, with Co-IP assay, we observed a direct protein interaction between TNFRSF14 and FAK in GBM cells (Fig. 4C). The subsequent experiment of truncation sequence plasmids confirmed a direct binding of the intracellular segment of TNFRSF14 to FAK (Fig. 4D, E and Figure S4E). Furthermore, transfection of FAK wild type overexpression vector in GBM cells obviously restored their reduced phosphate-FAK (p-FAK) and phosphate-p65 (p-p65) expression induced by TNFRSF14 knockdown, while FAK Y397F mutant vector couldn’t exert this role (Fig. 4F, G and Fig S4F). These data support FAK as a direct downstream effector of GBM intrinsic TNFRSF14 signaling. In addition, previous studies proved that FAK affected the activity of NF-κB subunit p65 by changing its nuclear and cytoplasmic distribution in malignant cells [24]. According to knockdown FAK by siRNA, we found that reduced FAK expression weakened p-FAK and decrease p-p65 expression in GBM cells (Figure S4G), and knockdown FAK inhibited nuclear translocation of p65 in these cells (Figure S4H). Then, we examined the expression of NF-κB pathway proteins including IκBα, phosphate-IκBα (p-IκBα), p65 and p-p65 in TNFRSF14 knockdown GBM cells. The data showed that the expression of p-IκBα and p-p65, not total IκBα and p65 was restrained with TNFRSF14 knockdown in GBM cells (Fig. 4B). This indicates ablation of TNFRSF14 in these cells inhibited the activity of NF-κB pathway (Fig. 4B). Besides, the incubation of Defactinib efficiently decreased the upregulation of NF-κB pathway proteins caused by TNFRSF14 over-expression (Figure S4D, p-IκBα and p-p65). Since p65 need to transport into the nucleus to perform its functions in transcription, we examined whether the distribution of p65 in GBM cells was affected by TNFRSF14. The result demonstrated that TNFRSF14 knockdown did affect nuclear translocation of p65 (Fig. 4H and I). The transfection of p65 wild type vector in TNFRSF14 knockdown GBM cells partially rescued p65 nuclear localization in these cells, which wasn’t observed in the cells transfected with nuclear localization sequence (NLS) mutant vector (Fig. 4J and Fig S4I). Furthermore, the reduced proliferation, migration and invasion of tumor cells induced by TNFRSF14 knockdown were partly restored by FAK wild type or p65 wild type vector transfection in these cells, which couldn’t be obtained by the transfection of FAK Y397F mutant and NLS mutant vectors (Fig. 4K-M). Altogether, these data indicate a cancer intrinsic TNFRSF14 signaling cascade in GBM cells, in which TNFRSF14 directly phosphorylates FAK at Y397 and thereby affects p65 nuclear translocation.

### Cancer intrinsic TNFRSF14 promotes the recruitment of TAMs through augmenting CXCL1 and CXCL5 secretion in GBM cells

**Fig. 5:**
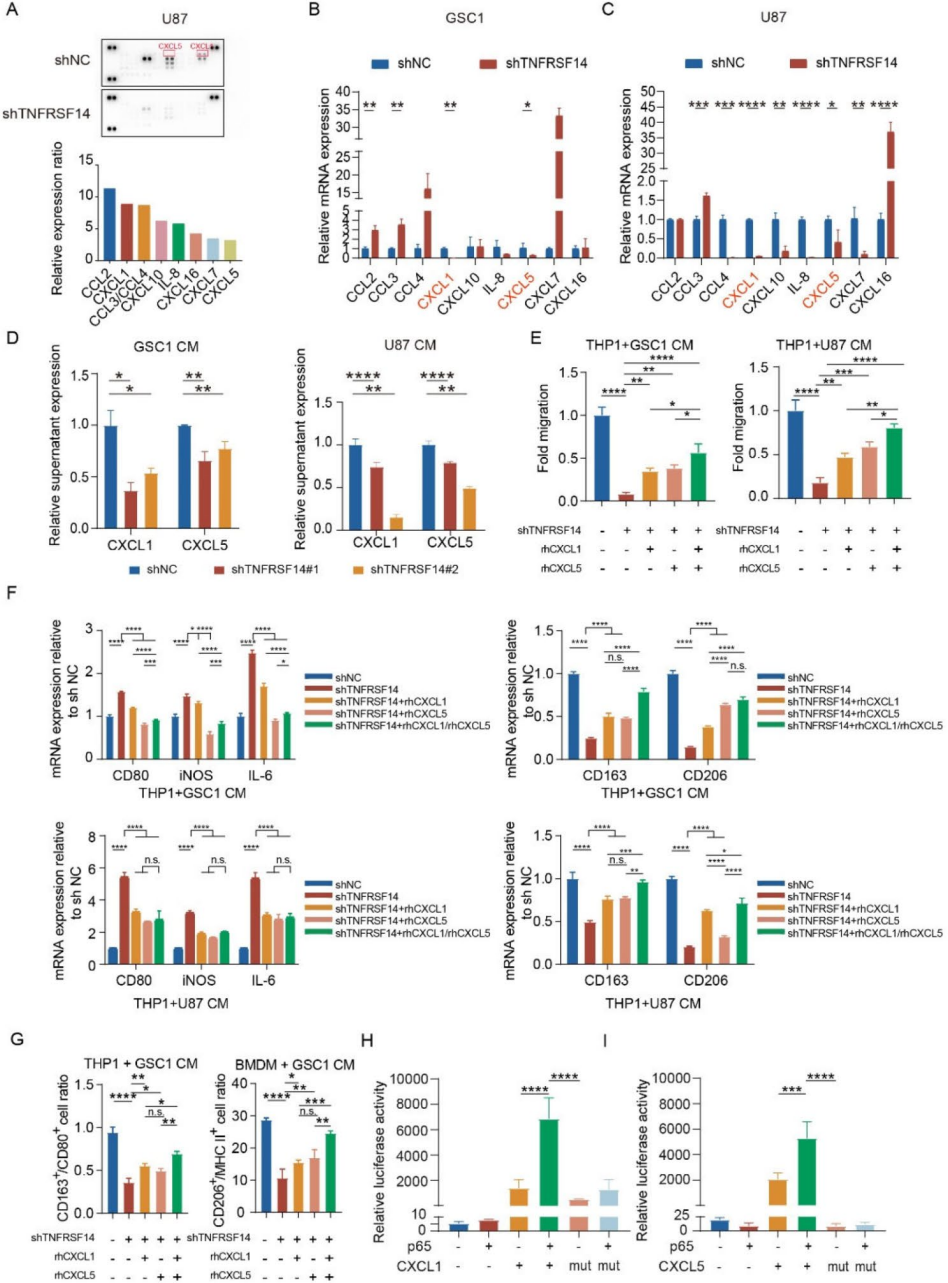
Glioma intrinsic TNFRSF14 promotes TAMs recruitment though a NF-κB dependent CXCL1 and CXCL5 secretin GBM cells. (**A**) Representative image (upper) and analyses (lower) of screening with human Cytokine Array in indicated GBM cells. (**B**, **C**) RT-PCR analyses of indicated cytokines in indicated GBM cells (relative to shNC, *n* = 3, one-way ANOVA). (**D**) ELISA of CXCL1 and CXCL5 in supernatant from indicated GBM cells (relative to shNC, *n* = 3, one-way ANOVA). (**E**) In vitro migration assay of THP1-derived macrophages incubated with CM from indicated GBM cells supplemented with rhCXCL1 and rhCXCL5 (relative to shNC, *n* = 3, one-way ANOVA). (**F**) qPCR and analysis of indicated markers in THP1-derived macrophages with indicated treatment (relative to shNC, *n* = 3, one-way ANOVA). (**G**) Flow cytometry analysis of indicated markers in THP1- and BMDM-derived macrophages with indicated treatment (relative to shNC, *n* = 3, one-way ANOVA). (**H**, **I**) Luciferase assay of NF-κB subunit p65 regulating CXCL1 (H) and CXCL5 (I) transcription (*n* = 3, one-way ANOVA). (n.s. *p* ≥ 0.05, * *p* < 0.05, ** *p* < 0.01, *** *p* < 0.001, **** *p* < 0.0001)

Cytokines serve as the vital intercellular signals from malignant cells to modulate non-tumor cell components in TME. Next, we utilized Human Cytokine Array Kit to screen potential cytokines involved in TNFRSF14 signaling cascade in GBM cells. CXCL1 and CXCL5 were revealed as two cytokines with significantly reduced expression at protein level after TNFRSF14 knockdown (Fig. 5A). This was further supported by the data obtained from qPCR analysis, which indicated that TNFRSF14 knockdown efficiently reduced CXCL1 and CXCL5 transcription (Fig. 5B and C). Moreover, ELISA demonstrated that TNFRSF14 knockdown strongly decreased the content of CXCL1 and CXCL5 in the culture supernatant from GBM cells (Fig. 5D). Given that CXCL1/CXCL5 were two well-known immunosuppressive cytokines and contributed to modulating TAMs behaviors [25], we next supplemented recombinant human CXCL1/CXCL5 (rhCXCL1/CXCL5) during the coculture of THP1- and BMDMs-derived macrophages with CM from TNFRSF14 knockdown GBM cells. Indeed, rhCXCL1/CXCL5 efficiently restored the decreased chemotaxis of THP1-derived macrophages induced by CM from TNFRSF14 knockdown GBM cells (Fig. 5E and Figure S5A). rhCXCL1/CXCL5 treatment induced elevated expression of CD163 and CD206, and reduced CD80, CD86 and IL-6 expression in THP-1 derived macrophages relative to the incubation of CM from TNFRSF14-knockdown GBM cells (Fig. 5F). Besides, the ratio of M2-type macrophages to M1-type macrophages were also decrease in THP-1 derived macrophages and BMDMs (Fig. 5G). However, the supplement of recombinant CXCL1 and CXCL5 could partially restore changes mentioned above (Fig. 5F and G). These observations suggest a potential cancer intrinsic TNFRSF14 mediating mechanism contributes to recruiting anti-inflammatory TAMs through CXCL1 and CXCL5 releasing from malignant cells.

Since our data proved that TNFRSF14 activates FAK/NF-κB axis to promote malignant phenotypes of GBM cells, we sought to examine whether TNFRSF14 affected CXCL1 and CXCL5 secretion in GBM cells through FAK/NF-κB activation. As expected, luciferase reporter assay confirmed the positive regulatory role of NF-κB subunit p65 to CXCL1 and CXCL5 transcription in GBM cells (Fig. 5H, I, Figure S5B and Table S7, S8). Altogether, these results suggest CXCL1 and CXCL5 are two major cytokines regulating GBM cell intrinsic TNFRSF14-induced macrophage phenotype transition, and these two cytokines are regulated by TNFRSF14/FAK/NF-κB axis in GBM cells.

### TNFRSF14 blockade attenuates GBM tumor growth, reshapes immunosuppressive microenvironment and improves therapeutic efficacy of anti-PD-L1 in GBM

**Fig. 6:**
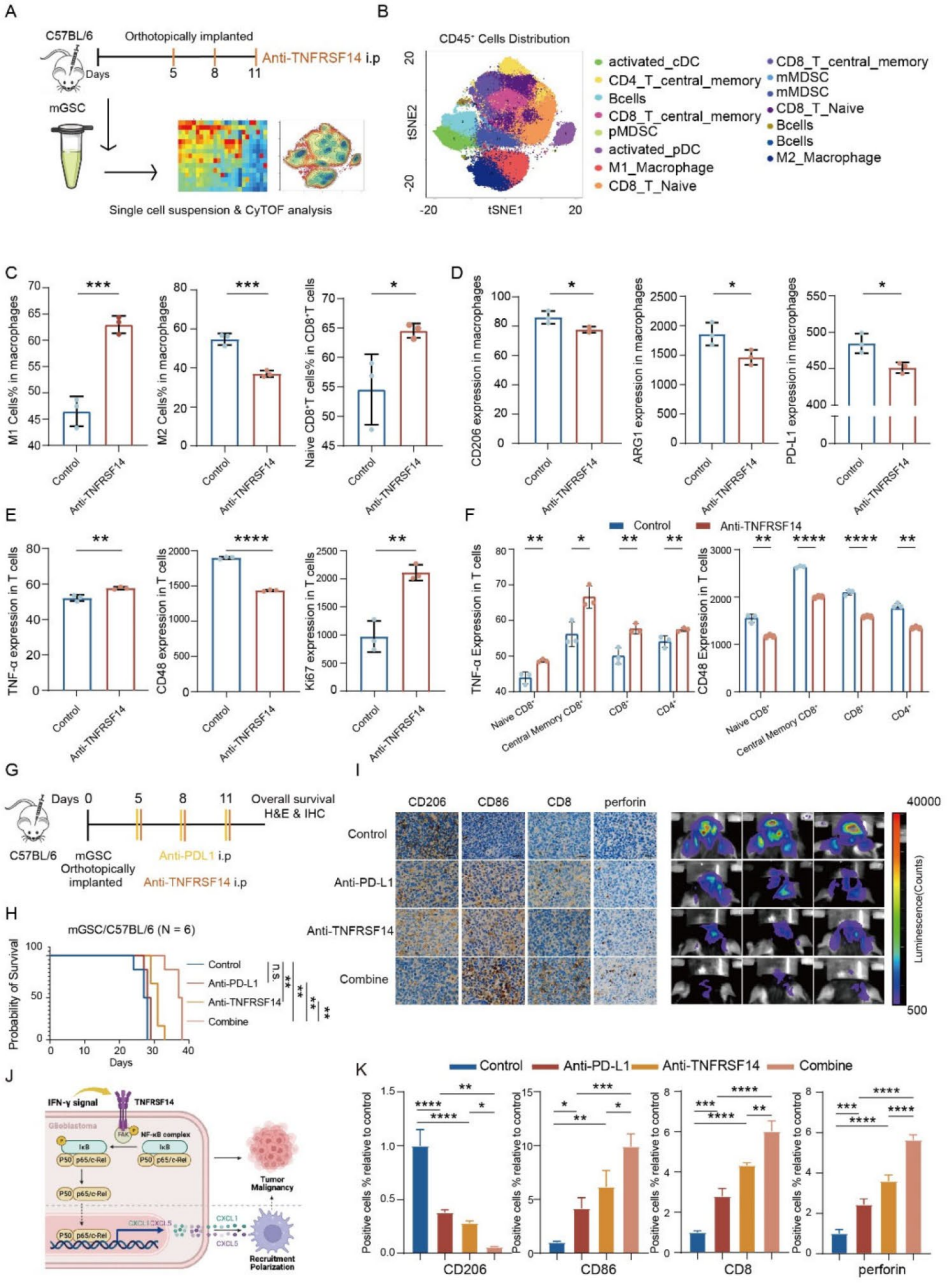
Blockade of TNFRSF14 impairs mice GBM growth and overcomes the resistance to anti-PD-L1. (**A**) Schematic graph of CyTOF analysis of anti-TNFRSF14 treatment and control samples from indicated mice GBM models. (**B**) The annotation of 15 cell clusters in CD45^+^ immune cells. (**C**-**E**) The expression analysis of indicated markers in macrophages and T cells subgroup under anti-TNFRSF14 and control treatment (C: left M1 cells represented by CD11b^+^F4/80^+^Ly-6G^−^CD3E^−^CD19^−^Ly-6C^−^CD80^+^CD86^+^ cells in macrophages; middle M2 cells represented by CD11b^+^F4/80^+^Ly-6G^−^CD3E^−^CD19^−^Ly-6C^−^ARG^+^CD206^+^ cells in macrophages; right Naïve CD8^+^ T cells represented by CD3^+^CD19^−^CD11b^−^NK1.1^−^TCRβ^+^TCRγδ^−^CD44^−^CD8^+^ cells in T cells; D: left: CD206 in macrophages, middle: ARG1 in macrophages, right PD-L1 in macrophages; E: left TNF-α in T cells, middle CD48 in T cells, right Ki67 in T cells) (*n* = 3, t-test). (**F**) The expression analysis of TNF-α and CD48 in indicated CD8^+^ and CD4^+^ T cell clusters) (*n* = 3, t-test). (**G**) Schematic graph of the administration of anti-TNFRSF14, anti-PD-L1 and combination treatment in indicated GBM mice model. (**H**) Survival analysis of mice intracranially transplanted mGSCs and then with indicated treatment (*n* = 6, log-rank). (**I**) Representative immunohistochemical staining of indicated markers (CD206, CD86, CD8 and perforin) and bioluminescence brain images of mice intracranially transplanted mGSCs and then with indicated treatment. (**J**) Graphic abstract of present study. (**K**) Analysis of staining in mice brain section shown in Fig. 6I (*n* = 3, one-way ANOVA). (n.s. *p* ≥ 0.05, * *p* < 0.05, ** *p* < 0.01, *** *p* < 0.001, **** *p* < 0.0001)

To further explore the role of TNFRSF14 in GBM, we harvested mouse GBM samples derived from mGSCs in immunocompetent mice model and performed CyTOF analysis (Fig. 6A). As shown in Fig. 6B, CD45^+^ immune cells were gated and 15 cell clusters were acquired for further analysis. We found that the ratio of M2-type macrophages (CD11b^+^F4/80^+^Ly-6G^−^CD3E^−^CD19^−^Ly-6C^−^ARG^+^CD206^+^ cells) decreased and M1-type macrophages (CD11b^+^F4/80^+^Ly-6G^−^CD3E^−^CD19^−^Ly-6C^−^CD80^+^CD86^+^ cells) increased in mice treated with Tnfrsf14 antibody (Fig. 6C), which was consistent with the results described above (Fig. 3B). Interestingly, we found an increased ratio of naive T cells in CD8 ^+^ T cells population in anti-Tnfrsf14 group (Fig. 6C), which indicates that the amelioration of T cell population status was started in tumors after the administration of Tnfrsf14 antibody. The expression of CD206, ARG1 and PD-L1 were downregulated in macrophages in anti-Tnfrsf14 group samples (Fig. 6D and Figure S6), while Ki67 and TNF-α were up-regulated in T cells in these samples. CD48, an immune checkpoint that has been reported associated with poor prognosis in gliomas [26], was also down-regulated in T cell population of anti-Tnfrsf14 group samples (Fig. 6E). In addition, the pattern of changes in TNF-α and CD48 expression was consistent across naïve CD8^+^, central memory CD8^+^, CD8^+^ and CD4^+^ T cells (Fig. 6F). These results demonstrate that TNFRSF14 blockade reversed the immunosuppressive microenvironment and initiated the anti-tumor immune process in GBM.

While anti-PD-L1 treatment induced compensatory TNFRSF14 elevation by IFN-γ production in mouse GBM tumor (Fig. 1I, K, and L), we sought to examine in vivo efficiency of TNFRSF14 inhibition and its combination with PD-L1 blockade on tumor growth in mouse immune competent orthotopic GBM model. Anti-PD-L1 (10 µg/g body weight) and anti-TNFRSF14 (5 µg/g body weight) antibodies were intraperitoneally injected into mice at the 5th, 8th, and 11th day after mGSCs and GL261 intracranial implantation (Fig. 6G and Figure S7A). In comparison to anti-TNFRSF14 mono-treatment, the combination of TNFRSF14 and PD-L1 blockade remarkably extend the survival of tumor-bearing mice (Fig. 6H and Figure S7B). Mice with TNFRSF14 antibody treatment, especially the combination treatment group, exhibited significantly decreased tumor growth and lower ratio of Ki67 staining positive cells (Fig. 6I and Figure S7C-F) in tumor tissues compared to control group and anti-PD-L1 mono-treatment group, respectively. Furthermore, IHC analysis in the above mGSCs or GL261 tumor specimens showed that TNFRSF14 antibody treatment led to less CD206^+^ and more CD86^+^ TAMs infiltration (Fig. 6I, K and Figure S7G, H). We also observed an elevation of CD8^+^ T cell presence and perforin expression, which were beneficial to restore anti-tumor immune response (Fig. 6I, K and Figure S7G, H). IHC analysis also confirmed that anti-TNFRSF14 treatment exactly reduced the expression of TNFRSF14 in mice GBM tumor, no matter compared with control group or PD-L1 blockade group (Figure S6I). Meanwhile, we didn’t observe significant sign of liver and kidney injury in TNFRSF14 antibody treatment with H&E analysis (Figure S7J). This supported further clinical safety evaluation for this strategy. Altogether, these results demonstrate that anti-TNFRSF14 treatment improved therapeutic efficacy of anti-PD-L1 in vivo and TNFRSF14 is a potential target for combination ICB strategy with anti-PD-L1 in GBM (Fig. 6J).

### The expression of TNFRSF14 and phosphorylation of FAK Y397 is positively associated with TAMs infiltration in GBM

**Fig. 7:**
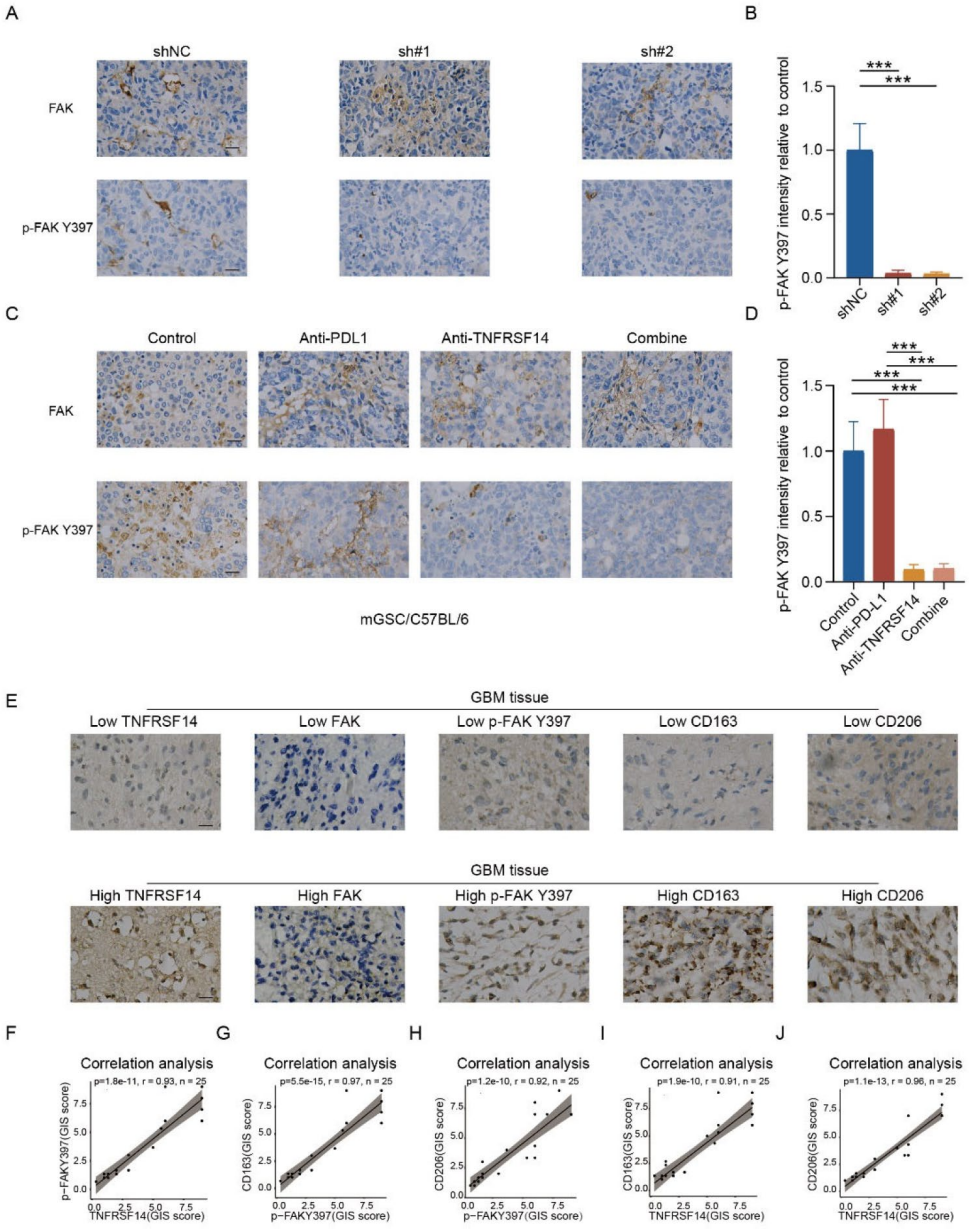
The expression of TNFRSF14 and phosphorylation of FAK Y397 is positively associated with TAMs infiltration in GBM. (**A**) Representative immunohistochemical staining of FAK and p-FAK Y397 in indicated mice brain section (scale bar, 25 μm). (**B**) Analysis of p-FAK Y397 staining intensities in Fig. 7A (*n* = 3, one-way ANOVA). (**C**) Representative immunohistochemical staining of FAK and p-FAK Y397 in indicated mice brain section after antibody administration (scale bar, 25 μm). (**D**) Analysis of p-FAK Y397 staining intensities in Fig. 7C (*n* = 3, one-way ANOVA). (**E**) Representative immunohistochemical staining images of TNFRSF14, FAK, p-FAK Y397, CD163 and CD206 in clinical GBM samples (*n* = 25). (**F**-**J**) Pearson’s correlation expression analysis of indicated markers (F: p-FAK Y397 and TNFRSF14; G: CD163 and p-FAK Y397; H: CD206 and p-FAK Y397; I: CD163 and TNFRSF14; J: CD206 and TNFRSF14) according to staining intensities evaluation based on GIS score in clinical GBM samples (*n* = 25). (n.s. *p* ≥ 0.05, *p < 0.05, ** *p* < 0.01, *** *p* < 0.001, **** *p* < 0.0001)

After observing the functional role of FAK/ FAK Y397 phosphorylation induced by cancer intrinsic TNFRSF14 in promoting malignant progression of GBM (Fig. 4), we sought to validate the association between FAK/ FAK Y397 phosphorylation induced by TNFRSF14 and TAMs infiltration in clinical GBM samples. We firstly examined the expression levels of FAK and p-FAK Y397 in tumor samples from mouse orthotopic GBM model (Figs. 2J and 6I). As expected, the phosphorylation level of FAK Y397 was significantly decreased after TNFRSF14 knockdown in vivo (Fig. 7A, B), and the level of p-FAK Y397 was also efficiently restrained by anti-TNFRSF14 mono-treatment or combined treatment with PD-L1 antibody (Fig. 7C, D). We also found a positive correlation between TNFRSF14 and p-FAK Y397 in clinical GBM specimens (*r* = 0.93, *p* < 0.0001, Fig. 7E, F). Besides, to validate the relationship between TNFRSF14/p-FAK Y397 and TAMs infiltration in GBM, we investigated the relationship between the expression of TNFRSF14/p-FAK Y397 and M2 TAMs markers (CD163 and CD206) by IHC staining in clinical GBM samples. Indeed, there was a significant positive correlation between the expression of p-FAK Y397 and M2 TAMs markers (CD163: *r* = 0.97, *p* < 0.0001, Fig. 7G; and CD206: *r* = 0.92, *p* < 0.0001, Fig. 7H) in GBM samples. Meanwhile, we observed a positive association between TNFRSF14 elevation and upregulation of M2 TAMs markers, CD163 (*r* = 0.91, *p* < 0.0001, Fig. 7I) and CD206 (*r* = 0.96, *p* < 0.0001, Fig. 7J) in GBM samples. Together, these data indicate that the expression of TNFRSF14 and phosphorylation of FAK Y397 is positively associated with TAMs infiltration in GBM.

## Discussion

Malignant intrinsic primary and adaptive resistance to therapeutics is a major obstacle in cancer treatment [27]. To escape therapeutics, cancer cells activate signaling pathways conducive to their survival, while releasing intercellular compensatory signals like cytokines to constitute TME facilitating their survival and escape immune surveillance. Here, we identify a novel cancer cell-intrinsic resistance mechanism conferring from IFN-γ signaling activation, mediated by TNFRSF14 and its downstream effector, FAK. TNFRSF14 is an immune checkpoint expressed by a variety of immune cells with co-stimulatory and co-inhibitory functions through binding with its ligands, TNFSF14 (LIGHT), to induce effective immune response. Alternatively, TNFRSF14 interacts with BTLA or CD160 to inhibit T cell immune response [28]. In cancer, TNFRSF14 upregulation is related to unfavorable survival of melanoma, gastric and colon cancer [29–32]. Its expression inversely correlated with the infiltration of CD4^+^, CD8^+^, CD45RO^+^ T lymphocytes in liver cancer and inhibits effective anti-tumor immunity [33]. A recent bioinformatic report reveals that TNFRSF14 elevation associated with poor prognosis in GBM and correlated with immunosuppression [18]. However, there is a lack of experimental validation. The functions and downstream effectors of TNFRSF14 remain undefined. In particular, the association between TNFRSF14 and cancer cell IFN-γ signaling activation and the cell populations in which TNFRSF14 exerts biological effect in GBM remains to be further clarified. In present work, we demonstrate that TNFRSF14 facilitates the phosphorylation of FAK at Y397 in GBM cells. The activation of TNFRSF14/FAK signaling subsequently activates NF-κB complex and promotes the nuclear translocation of p65, leading to the enhanced tumorigenicity of GBM cells, as well as increasing their CXCL1 and CXCL5 secretion, which facilitates the constitution of immunosuppressive TME through recruiting anti-inflammatory TAMs.

IFN-γ signaling activation was an indicator of the effectiveness of immunotherapy. However, growing evidences disclosed dual role of IFN-γ signaling activation in cancer biology [9, 21, 34, 35]. While activating effective immune response, IFN-γ sensing program in malignant cells protects them from cytotoxic effect induced by IFN-γ and promotes cancer progression. IFN-γ from cytotoxic T cells is a prominent driver for effective ICB response in cancer [4]. However, IFN-γ exposure leads to JAK/STAT activation which elevates the expression of IFN-stimulating genes (ISGs) and multiple T cell inhibitory receptors (TCIR) ligands in cancer cells. This inhibits the effective immune response induced by IFN-γ and leads to adaptive resistance to ICB and even cancer hyper-progression during ICB [3, 6, 7]. In addition, cancer cell ISGs upregulation has been indicated as the predictor of therapeutic resistance to radiation and chemotherapy [3, 36]. These observations indicate complicated roles of IFN-γ signaling activation in cancer.

Previously, we disclosed the involvement of IFN signaling activation contributing to GBM progression [8], which reflects the potential resistance mechanisms of GBM cells to therapeutics through activating IFN signaling. Considering that cancer cells may activate alternative immune suppressive molecules to acquire the resistance to IFN induced by ICB treatment, we sought to search for the ICs most significantly associated with IFN signaling activation in GBM, which may provide new therapeutic target for this destructive tumor. Through screening, TNFRSF14 was identified as the candidate for further investigation, which have both of cancer intrinsic and non-tumor cell expression in GBM. The subsequent expression validation identified a higher TNFRSF14 elevation than PD-L1 in GBM cells after IFN-γ treatment, instead of IFN-α or IFN-β exposure, which complied with a dose-dependent manner. TNFRSF14 was previously demonstrated as one of T cell inhibitory receptor (TCIR) ligands associated with ICB resistance through JAK/STAT1/interferon-γ stimulating gene signaling activation [6]. However, the current characterization on its role as a compensatory IC inducing tumor resistance to immunotherapy remains quite limited. Our study provides direct evidences for the association between TNFRSF14 upregulation and IFN-γ signaling activation in TME: IFN-γ induces a compensatory TNFRSF14 elevation in GBM cells, and it may be a more suitable target than PD-L1 for GBM immunotherapy. Notably, we observed that PD-L1 blockade led to IFN–γ elevation in mouse immune competent GBM tumor, while inducing TNFRSF14 upregulation in these samples. These findings delineate a novel acquired cancer resistance mechanism to anti-PD-L1: PD-L1 blockade induced TNFRSF14 elevation in malignant cells, which serves as the dominant IC mediating cancer intrinsic adaptative resistance to PD-L1 blockade and related IFN-γ elevation in GBM. The administration of anti-TNFRSF14 efficiently improves the sensitivity of GBM cells to anti-PD-L1, and the combined blockade of PD-L1 and TNFRSF14 significantly reduces GBM progression in mouse immune competent model. TNFRSF14 serves as a crucial compensatory mechanism for the acquired resistance of GBM cells to IFN-γ and anti-PD-L1 treatment, and combination blockade of TNFRSF14 and PD-L1 brings improved therapeutic benefits than anti-PD-L1 monotherapy in GBM.

Another interesting finding in current study is the validation of direct TNFRSF14/FAK interaction. It has been indicated that TNFRSF14 may interact with FAK [37, 38], a non-receptor tyrosine protein kinase, which is well-known for its role in tumor initiation and progression and promoting the survival and immune evasion of cancer cells through kinase-dependent and kinase-independent mechanisms [37, 39–41]. However, there is no direct verification. To our knowledge, we provide the first experimental evidence for their interaction: TNFRSF14 phosphorylates FAK at Y397, which activates downstream NF-κB signaling through promoting nuclear translocation of p65. In addition, Defactinib, a FAK inhibitor, efficiently reduces in vitro GBM cell proliferation brought by cancer intrinsic TNFRSF14 elevation. In vivo study is needed to further support the potential clinical application of Defactinib.

As the most abundant immune cell component in GBM, TAMs have been reported to facilitate GBM progression by contributing to the constitution of immunosuppressive TME [42, 43]. While IFN-γ sensing program induces TNFRSF14 upregulation in tumor cells, this cancer intrinsic TNFRSF14 elevation augments the recruitment of anti-inflammatory TAMs in preclinical mouse GBM model. Then, according to the macrophage chemokine array screening, CXCL1 and CXCL5 were revealed as two dominant downstream chemokines of malignant intrinsic TNFRSF14/FAK signaling, which contributed to recruiting TAMs. GBM cells not only release CXCL1 and CXCL5 to recruit TAMs, but switch them into anti-inflammatory phenotype. Ablating cancer intrinsic TNFRSF14 alters TME constitution with elevated perforin expression and more CD8^+^ T cell infiltration accompanying with less anti-inflammatory macrophages. Indeed, combination of TNFRSF14 and PD-L1 blockade exhibited a satisfied therapeutic efficacy in mouse immune competent preclinical model without obvious hepatorenal toxicity. This supports TNFRSF14 as a promising therapeutic target for GBM. Further investigation on the side effects of systemic TNFRSF14 blockade will be conducive to the clinical translation of this immunotherapeutic strategy.

Collectively, to our knowledge, the present work is the first study giving detailed functional and mechanistic insights on immune checkpoint TNFRSF14 induced by IFN-γ signaling activation. Our study highlights a novel resistant mechanism to IFN exposure and PD-L1 blockade in cancer: Malignant cells under IFN-γ exposure in TME acquire intrinsic TNFRSF14 elevation, which leads to resistance to ICB. Mechanistically, cancer intrinsic TNFRSF14 phosphorylates FAK at Y397 and activates its downstream NF-κB signaling, which not only enhances the tumorigenicity of GBM cells, but promotes the recruitment of anti-inflammatory TAMs through elevating CXCL1 and CXCL5 secretion from GBM cells. TNFRSF14 is a potential suitable target for developing new ICB strategies in malignant glioma, and the combination of TNFRSF14 blockade with anti-PD-L1 provides synergic benefit for GBM.

## Conclusion

Our findings suggest that although ICB improves immune responses to cancer, malignant cells could develop therapy resistance by intrinsically elevating alterative ICs through IFN-γ sensing program, which leads to the failure of ICB. The identification of context-dependent key compensatory ICs is important for the improvement of current ICB strategies. Targeting the adaptive activated IC in the context of cancer immunotherapy, and carrying out combined strategy may be an effective way to improve cancer response to ICB treatment in clinics.

### Electronic supplementary material

Below is the link to the electronic supplementary material.

Supplementary Material 1

## Data Availability

The experimental data presented in the study are included in the article/ Supplementary Materials, and further inquiries will be directed to the corresponding authors upon reasonable request.

## Abbreviations

TNFRSF14: TNF receptor superfamily member 14
IFN: Interferon
GBM: Glioblastoma
IC: Immune checkpoint
ICB: Immune checkpoint blockade
TAM: Tumor associated macrophage
ICD: Immunogenic cell death
TME: Tumor microenvironment
BMDM: Bone marrow-derived macrophage
NLS: Nuclear localization sequence
CyTOF: Cytometry by time-of-flight mass cytometry
IHC: Immunohistochemistry
Co-IP: Co-immunoprecipitation
ELISA: Enzyme-linked immunosorbent assay
RT-qPCR: Real-time quantitative polymerase chain reaction
