# Supplementary material for: Inhibiting interferon-γ induced cancer intrinsic TNFRSF14 elevation restrains the malignant progression of glioblastoma
Source: J Exp Clin Cancer Res. 2024 Jul 31;43:212. doi: 10.1186/s13046-024-03131-7 (PMC11289992; doi:10.1186/s13046-024-03131-7)
Supplement: Supplementary file 1 — Supplementary Material 1 [file 13046_2024_3131_MOESM1_ESM.docx]

**Supplementary information**

**This file includes:**

**Supplementary Tables and Unprocessed original images of gels in this article**

**1. Supplementary Tables**

Table S1. The information of reagents employed in present study.

Table S2. Specific sequences of lentiviral shRNA and siRNA employed in the present study.

Table S3. Target gene symbols and corresponding primer sequences of qRT-PCR.

Table S4. The list of Immune checkpoints employed for analysis in Figure 1A.

Table S5. CGGA, TCGA and CMU sample information.

Table S6. Correlation analysis of immune checkpoints and type I, type II IFN signaling activation in GBM (CGGA: n = 144; TCGA: n = 168; and CMU dataset: n = 208).

Table S7. Potential transcription factor binding sites for p65 in CXCL1 promoter predicted by JASPAR.

Table S8. Potential transcription factor binding sites for p65 in CXCL5 promoter predicted by JASPAR.

**Table S1. The information of reagents employed in present study.**

| **REAGENT or RESOURCE** | | **SOURCE** | | | **IDENTIFIER** |
| --- | --- | --- | --- | --- | --- |
| **Antibodies** |  | | |  | |
| IF: GFAP |  | Proteintech | | | 16825-1-AP |
| IF: PDL1 |  | Abcam | | | #ab213480 |
| IF&WB: TNFRSF14 |  | Abcam | | | #ab62462 #ab47677 |
| IF&WB: FAK |  | Cellsignalingtech. | | | #71433 |
| WB: β-ACTIN |  | Abcam | | | #ab8226 |
| WB: Phospho-FAK (Tyr397) |  | Cellsignalingtech. | | | *#*8556 |
| WB: Phospho-NF-κB p65 (Ser536) |  | Cellsignalingtech. | | | *#*3033 |
| WB&IF: NF-κB p65 |  | Cellsignalingtech. | | | #8242 |
| WB: FLAG |  | Cellsignalingtech. | | | #14793 |
| WB: p-IKBα |  | Cellsignalingtech. | | | #9246 |
| WB: IKBα |  | Cellsignalingtech. | | | #4814 |
| WB: MYC |  | Proteintech | | | 16286-1-AP |
| WB: GST |  | Proteintech | | | HRP-66001 |
| WB: HIS |  | Proteintech | | | 66005-1-Ig |
| WB: GAPDH |  | Proteintech | | | 60004-1-Ig |
| WB: HistoneH3 |  | Proteintech | | | 68345-1-Ig |
| WB&IHC: IFN-gamma |  | ebioscience | | | MM700B |
| IHC: CXCL5 |  | ebioscience | | | PA5-115069 |
| IHC: CXCL1 |  | Proteintech | | | 12335-1-AP |
| IHC: CD8 |  | Proteintech | | | 66868-1-Ig |
| IHC: ki67 |  | Proteintech | | | 27309-1-AP |
| IHC: CD3 |  | Proteintech | | | 17617-1-AP |
| IHC: IBA1 |  | Abcam | | | #ab178846 |
| IHC: CD86 |  | Abcam | | | #ab220188 |
| IHC: CD206 |  | Proteintech | | | 60143-1-Ig |
| IHC: Perforin |  | Cellsignalingtech. | | | #31647 |
| Flow: CD45 |  | BD Pharmingen | | | #563891 |
| Flow: CD11b |  | BD Pharmingen | | | #557000 |
| Flow: F4/80 |  | BD Pharmingen | | | #565411 |
| Flow: MHCII |  | BD Pharmingen | | | #557000 |
| Flow: CD206 |  | R&D Systems | | | FAB25351R |
| Flow: CD3e |  | BD Pharmingen | | | #551163 |
| Flow: CD4 |  | BD Pharmingen | | | #552051 |
| Flow: CD8a |  | BD Pharmingen | | | #553030 |
| Flow: IFN-γ |  | BD Pharmingen | | | #554412 |
| Flow: TNF-α |  | BD Pharmingen | | | #554420 |
| In vivo: TNFRSF14 |  | ebioscience | | | 16-5962-38 |
| In vivo: PDL1 |  | BioXcell | | | BE0101 |
| **Biological samples** |  | |  | |  |
| Individual human glioma tissue samples |  | China medical University | | | NA |
| **Chemicals, Peptides, and Recombinant Proteins** |  |  | | |  |
| Defactinib |  | MedChemExpress | | | HY-12289 |
| DAPI |  | Solarbio | | | C0060 |
| Recombinant IFN-γ |  | Proteintech | | | HZ-1301 |

**Table S2. Specific sequences of lentiviral shRNA and siRNA employed in the present study.**

| **Species** | **NO.** | **Sequences** | **GC%** |
| --- | --- | --- | --- |
| Human | TNFRSF14-RNAi(sh#1) | tgGAGGAGACAATACCCTCAT | 47.37% |
| Human | TNFRSF14-RNAi(sh#2) | gcTGGTGCTGTATCTCACCTT | 47.37% |
| Mouse | Tnfrsf14-RNAi(sh#1) | gcATTTCAACAGGAAGTAAGA | 31.58% |
| Mouse | Tnfrsf14-RNAi(sh#2) | ccACAGACATATACCGCCCAT | 47.37% |
| Human | si-FAK-1 | GAUGUUGGUUUAAAGCGAUUUTT | 30.43% |
| Human | si-FAK-2 | CAACAGGUGAAGAGCGAUUAUTT | 39.13% |

**Table S3.** **Target gene symbols and primer sequences of qRT-PCR.**

| Gene | | Primer sequence |
| --- | --- | --- |
| Human TNFRSF14 | Forward | TTCTCTCAGGGAGCCTCGTCAT |
|  | Reverse | CTCACCTTCTGCCTCCTGTCTT |
| Mouse TNFRSF14 | Forward | CCAGGCTACTTCTGTGAGAACC |
|  | Reverse | CAGTCAGCACATACAGTGTCCTG |
| CCL2 | Forward | AGAATCACCAGCAGCAAGTGTCC |
|  | Reverse | TCCTGAACCCACTTCTGCTTGG |
| CCL3 | Forward | ACTTTGAGACGAGCAGCCAGTG |
|  | Reverse | TTTCTGGACCCACTCCTCACTG |
| CCL4 | Forward | GCTTCCTCGCAACTTTGTGGTAG |
|  | Reverse | GGTCATACACGTACTCCTGGAC |
| CXCL1 | Forward | AGCTTGCCTCAATCCTGCATCC |
|  | Reverse | TCCTTCAGGAACAGCCACCAGT |
| CXCL10 | Forward | GGTGAGAAGAGATGTCTGAATCC |
|  | Reverse | GTCCATCCTTGGAAGCACTGCA |
| IL8 | Forward | GAGAGTGATTGAGAGTGGACCAC |
|  | Reverse | CACAACCCTCTGCACCCAGTTT |
| CXCL5 | Forward | CAGACCACGCAAGGAGTTCATC |
|  | Reverse | TTCCTTCCCGTTCTTCAGGGAG |
| CXCL7 | Forward | TGCTCTGGCTTCCTCCACCAAA |
|  | Reverse | ACACATGCAGCGGAGTTCAGCA |
| CXCL16 | Forward | CCTATGTGCTGTGCAAGAGGAG |
|  | Reverse | CTGGGCAACATAGAGTCCGTCT |
| Human CD80 | Forward | CTCTTGGTGCTGGCTGGTCTTT |
|  | Reverse | GCCAGTAGATGCGAGTTTGTGC |
| Human iNOS | Forward | GCTCTACACCTCCAATGTGACC |
|  | Reverse | CTGCCGAGATTTGAGCCTCATG |
| Human IL-6 | Forward | AGACAGCCACTCACCTCTTCAG |
|  | Reverse | TTCTGCCAGTGCCTCTTTGCTG |
| Human CD163 | Forward | CCAGAAGGAACTTGTAGCCACAG |
|  | Reverse | CAGGCACCAAGCGTTTTGAGCT |
| Human CD206 | Forward | AGCCAACACCAGCTCCTCAAGA |
|  | Reverse | CAAAACGCTCGCGCATTGTCCA |
| Human 18S | Forward | ACCCGTTGAACCCCATTCGTGA |
|  | Reverse | GCCTCACTAAACCATCCAATCGG |
| Mouse CD80 | Forward | CCTCAAGTTTCCATGTCCAAGGC |
|  | Reverse | GAGGAGAGTTGTAACGGCAAGG |
| Mouse CD86 | Forward | ACGTATTGGAAGGAGATTACAGCT |
|  | Reverse | TCTGTCAGCGTTACTATCCCGC |
| Mouse IL-1beta | Forward | TGGACCTTCCAGGATGAGGACA |
|  | Reverse | GTTCATCTCGGAGCCTGTAGTG |
| Mouse CD163 | Forward | GGCTAGACGAAGTCATCTGCAC |
|  | Reverse | CTTCGTTGGTCAGCCTCAGAGA |
| Mouse CD206 | Forward | GTTCACCTGGAGTGATGGTTCTC |
|  | Reverse | AGGACATGCCAGGGTCACCTTT |
| Mouse GAPDH | Forward | CATCACTGCCACCCAGAAGACTG |
|  | Reverse | ATGCCAGTGAGCTTCCCGTTCAG |

**Table S4. The list of Immune checkpoints employed for analysis in Figure 1A.**

| No. | Immune checkpoints |  | No. | Immune checkpoints |  | No. | Immune checkpoints |
| --- | --- | --- | --- | --- | --- | --- | --- |
| 1 | BTLA |  | 24 | CD86 |  | 47 | TNFRSF10C |
| 2 | BTN1A1 |  | 25 | CD96 |  | 48 | TNFRSF10D |
| 3 | BTN2A1 |  | 26 | CRTAM |  | 49 | TNFRSF11A |
| 4 | BTN2A2 |  | 27 | CTLA4 |  | 50 | TNFRSF11B |
| 5 | BTN2A3P |  | 28 | HAVCR1 |  | 51 | TNFRSF12A |
| 6 | BTN3A1 |  | 29 | HAVCR2 |  | 52 | TNFRSF13B |
| 7 | BTN3A2 |  | 30 | HHLA2 |  | 53 | TNFRSF13C |
| 8 | BTN3A3 |  | 31 | ICOS |  | 54 | TNFRSF14 |
| 9 | BTNL2 |  | 32 | LGALS9 |  | 55 | TNFRSF17 |
| 10 | BTNL3 |  | 33 | LTA |  | 56 | TNFRSF18 |
| 11 | BTNL8 |  | 34 | LY9 |  | 57 | TNFRSF19 |
| 12 | BTNL9 |  | 35 | NCR3LG1 |  | 58 | TNFRSF1A |
| 13 | CD160 |  | 36 | PDCD1 |  | 59 | TNFRSF1B |
| 14 | CD226 |  | 37 | PDCD1LG2 |  | 60 | TNFRSF21 |
| 15 | CD244 |  | 38 | PVR |  | 61 | TNFRSF25 |
| 16 | CD27 |  | 39 | RTEL1-TNFRSF6B |  | 62 | TNFRSF4 |
| 17 | CD274 |  | 40 | SLAMF1 |  | 63 | TNFRSF8 |
| 18 | CD276 |  | 41 | SLAMF6 |  | 64 | TNFRSF9 |
| 19 | CD28 |  | 42 | SLAMF8 |  | 65 | TNFSF14 |
| 20 | CD40 |  | 43 | SLAMF9 |  | 66 | TNFSF4 |
| 21 | CD48 |  | 44 | TIGIT |  | 67 | VTCN1 |
| 22 | CD80 |  | 45 | TNFRSF10A |  |  |  |
| 23 | CD84 |  | 46 | TNFRSF10B |  |  |  |

**Table S5. CGGA, TCGA and CMU sample information.**

| **Database** |  | **CGGA** | **TCGA** | **CMU** |
| --- | --- | --- | --- | --- |
| Sequencing platform |  | Illumina Hiseq 2000 RNAseq | Illumina Hiseq 2000 RNAseq | Illumina Hiseq 2000 RNAseq |
| Total |  | 325 | 699 | 290 |
| Age | Average (range) | 43.32（8-81） | 47.34 (14-89) | 68.7(21-84) |
|  | NA | 0 | 63 | 102 |
| Sex | Male | 233 | 268 | 115 |
|  | Female | 122 | 368 | 73 |
|  | NA | 0 | 63 | 102 |
| KPS score | Average (range) | 76.25  （20-100） | 84.29  (40-100) | 67.5  (0-100) |
|  | NA | 0 | 305 | 102 |
| Primary/  recurrent | Primary | 241 | 619 | 130 |
|  | Recurrent | 84 | 0 | 58 |
|  | NA | 0 | 80 | 102 |
| Grade | II | 109 | 223 | 68 |
|  | III | 72 | 245 | 14 |
|  | GBM | 144 | 168 | 208 |
|  | NA | 0 | 63 | 0 |
| IDH (GBM) | Wild | 96 | 149 | 165 |
|  | Mutant | 32 | 12 | 10 |
|  | NA | 16 | 7 | 115 |

**Table S6. Correlation analysis of immune checkpoints and type I, type II IFN signaling activation in GBM (CGGA: n = 144; TCGA: n = 168; and CMU dataset: n = 208).**

| type I | | | | | |  | type II | | | | | |
| --- | --- | --- | --- | --- | --- | --- | --- | --- | --- | --- | --- | --- |
| CGGA (P < 0.001) | | TCGA (P < 0.001) | | CMU (P < 0.001) | |  | CGGA (P < 0.001) | | TCGA (P < 0.001) | | CMU (P < 0.001) | |
| Gene | r | Gene | r | Gene | r |  | Gene | r | Gene | r | Gene | r |
| PDCD1LG2 | 0.6787 | BTN3A3 | 0.6872 | LGALS9 | 0.8372 |  | PDCD1LG2 | 0.7293 | CD86 | 0.6736 | CD226 | 0.7986 |
| CD48 | 0.6682 | CD48 | 0.6750 | CD86 | 0.8268 |  | TNFRSF14 | 0.7070 | PDCD1LG2 | 0.6301 | TNFRSF14 | 0.7020 |
| CRTAM | 0.6366 | LGALS9 | 0.6687 | CD48 | 0.8140 |  | TNFRSF1B | 0.6604 | BTN3A3 | 0.6185 | BTN2A2 | 0.6796 |
| HAVCR2 | 0.6308 | BTN3A1 | 0.6677 | HAVCR2 | 0.7964 |  | HAVCR2 | 0.6200 | HAVCR2 | 0.6182 | TNFRSF1A | 0.6615 |
| CD27 | 0.6299 | CD86 | 0.6493 | CD84 | 0.7645 |  | TNFRSF1A | 0.6178 | CD48 | 0.6178 | BTN2A1 | 0.6560 |
| CD86 | 0.5886 | HAVCR2 | 0.6173 | SLAMF6 | 0.7279 |  | SLAMF8 | 0.6153 | BTN3A1 | 0.5999 | TNFRSF1B | 0.6446 |
| TNFRSF14 | 0.5769 | CD80 | 0.6170 | CD96 | 0.7108 |  | CD86 | 0.6122 | TNFRSF14 | 0.5923 | CD48 | 0.6020 |
| PDCD1 | 0.5734 | BTN3A2 | 0.5757 | ICOS | 0.6966 |  | CD48 | 0.6059 | LGALS9 | 0.5919 | TNFRSF10B | 0.5801 |
| TNFRSF1B | 0.5693 | PDCD1LG2 | 0.5696 | TNFRSF1B | 0.6817 |  | PVRL2 | 0.5872 | TNFRSF1A | 0.5796 | TNFRSF12A | 0.5632 |
| ICOS | 0.5568 | TNFRSF14 | 0.5666 | LY9 | 0.6790 |  | PDCD1 | 0.5715 | CD80 | 0.5708 | SLAMF1 | 0.5554 |
| CD96 | 0.5483 | C10orf54 | 0.5042 | SLAMF1 | 0.6743 |  | TNFRSF12A | 0.5705 | BTN3A2 | 0.5614 | TNFRSF10A | 0.5536 |
| LGALS9 | 0.5325 | CRTAM | 0.4896 | TNFRSF14 | 0.6735 |  | TNFRSF10A | 0.5595 | TNFRSF1B | 0.5564 | SLAMF8 | 0.5462 |
| SLAMF8 | 0.5302 | CD96 | 0.4881 | CTLA4 | 0.6367 |  | CD40 | 0.5526 | SLAMF8 | 0.5077 | CRTAM | 0.5417 |
| BTN3A2 | 0.5232 | TNFRSF1B | 0.4652 | ISG20 | 0.6292 |  | CD27 | 0.5358 | C10orf54 | 0.4870 | CTLA4 | 0.5284 |
| CD80 | 0.5064 | ICOS | 0.4501 | BTN3A3 | 0.6150 |  | CRTAM | 0.5313 | CD96 | 0.4832 | CD80 | 0.5251 |
| BTN3A3 | 0.4977 | SLAMF8 | 0.4453 | TIGIT | 0.6121 |  | CD226 | 0.5169 | CRTAM | 0.4806 | TNFRSF21 | 0.4966 |
| CD244 | 0.4616 | CD40 | 0.4440 | CD80 | 0.5769 |  | BTN3A2 | 0.5164 | CD40 | 0.4747 | BTN3A1 | 0.4313 |
| TNFRSF1A | 0.4496 | SLAMF6 | 0.4430 | PDCD1LG2 | 0.5705 |  | CD96 | 0.5108 | SLAMF6 | 0.4610 | CD40 | 0.4189 |
| CD226 | 0.4478 | CD27 | 0.4109 | BTN2A2 | 0.5550 |  | ICOS | 0.4895 | CD226 | 0.4438 | TIGIT | 0.4078 |
| SLAMF1 | 0.4426 | CD244 | 0.4105 | IRF9 | 0.5541 |  | TNFRSF10C | 0.4867 | ICOS | 0.4389 | TNFRSF10C | 0.3974 |
| TNFRSF10A | 0.4386 | SLAMF1 | 0.4101 | TNFRSF10C | 0.5191 |  | LGALS9 | 0.4812 | TNFRSF11A | 0.4215 | BTN3A2 | 0.3934 |
| C10orf54 | 0.4377 | TNFRSF1A | 0.4068 | BTN3A2 | 0.5072 |  | CD80 | 0.4769 | SLAMF1 | 0.4159 | PDCD1LG2 | 0.3876 |
| TIGIT | 0.4274 | LY9 | 0.3989 | PDCD1 | 0.5008 |  | LY9 | 0.4525 | TNFRSF10C | 0.4130 | TNFRSF6B | 0.3839 |
| PVRL2 | 0.4223 | CD226 | 0.3940 | NLRC5 | 0.4897 |  | SLAMF1 | 0.4422 | TNFRSF11B | 0.3984 | CD274 | 0.3797 |
| CD40 | 0.4198 | CD84 | 0.3766 | TNFRSF10A | 0.4824 |  | C10orf54 | 0.4215 | CD244 | 0.3945 | TNFRSF9 | 0.3748 |
| LY9 | 0.4132 | PDCD1 | 0.3606 | IFIT5 | 0.4802 |  | CD244 | 0.3885 | LY9 | 0.3900 | CD28 | 0.3715 |
| TNFRSF10C | 0.3601 | TIGIT | 0.3435 | CD40 | 0.4760 |  | TNFRSF10D | 0.3877 | CD27 | 0.3822 | CD27 | 0.3701 |
| TNFRSF11A | 0.3515 | BTN2A2 | 0.3429 | CD28 | 0.4715 |  | **TNFSF14** | **0.3855** | CD84 | 0.3660 | PDCD1 | 0.3564 |
| TNFRSF17 | 0.3306 | TNFRSF11B | 0.3299 | CD27 | 0.4703 |  | TNFSF4 | 0.3701 | TNFRSF10A | 0.3437 | TNFRSF18 | 0.3490 |
| CD28 | 0.3281 | TNFRSF11A | 0.3217 | BTN3A1 | 0.4676 |  | TNFRSF11A | 0.3633 | CD274 | 0.3400 | CD84 | 0.3486 |
|  | | TNFRSF10C | 0.3057 | TNFRSF1A | 0.4410 |  | BTN3A3 | 0.3491 | PVRL2 | 0.3354 | TNFRSF4 | 0.3315 |
|  |  |  | | SLAMF8 | 0.4309 |  | TNFRSF10B | 0.3456 | BTN2A2 | 0.3240 | **TNFSF14** | **0.3238** |
|  |  |  |  | TNFSF14 | 0.4240 |  | TNFRSF9 | 0.3439 | PDCD1 | 0.3185 | CD86 | 0.3186 |
|  |  |  |  | CD244 | 0.4011 |  | TNFRSF18 | 0.3372 | TNFRSF6B | 0.3180 |  | |
|  |  |  |  | CD226 | 0.3957 |  | SLAMF9 | 0.3330 | CD28 | 0.3095 |  |  |
|  |  |  |  | CRTAM | 0.3771 |  | TNFRSF11B | 0.3283 | TIGIT | 0.3041 |  |  |
|  |  |  |  | TNFRSF11A | 0.3642 |  | TIGIT | 0.3257 |  | | | |

**Table S7. Potential transcription factor binding sites for p65 in CXCL1 promoter predicted by JASPAR.**

| Matrix ID | Name | Score | Relative score | Start-End | Strand | Predicted sequence |
| --- | --- | --- | --- | --- | --- | --- |
| MA0107.1 | MA0107.1.RELA | 15.26936 | 0.999999999 | 1922-1931 | + | GGGAATTTCC |
| MA0107.1 | MA0107.1.RELA | 11.98946 | 0.914170511 | 1947-1956 | + | CGGGCTTTCC |
| MA0107.1 | MA0107.1.RELA | 8.406022 | 0.820397913 | 859-868 | + | CTGAAATTCC |

**Table S8. Potential transcription factor binding sites for p65 in CXCL5 promoter predicted by JASPAR.**

| Matrix ID | Name | Score | Relative score | Start-End | Strand | Predicted sequence |
| --- | --- | --- | --- | --- | --- | --- |
| MA0107.1 | MA0107.1.RELA | 15.26936 | 0.999999999 | 1910-1919 | + | GGGAATTTCC |
| MA0107.1 | MA0107.1.RELA | 9.43913 | 0.8474326 | 1935-1944 | + | TTGAGTTTCC |
| MA0107.1 | MA0107.1.RELA | 8.563163 | 0.824510021 | 1307-1316 | + | CGGACTTACC |
| MA0107.1 | MA0107.1.RELA | 8.403238 | 0.820325067 | 423-432 | + | TGAAGTTTCC |
| MA0107.1 | MA0107.1.RELA | 7.970129 | 0.808991315 | 414-423 | + | GTGAATTTCT |

**2.Unprocessed original images of gels in this article**

**Source of Fig 1B**
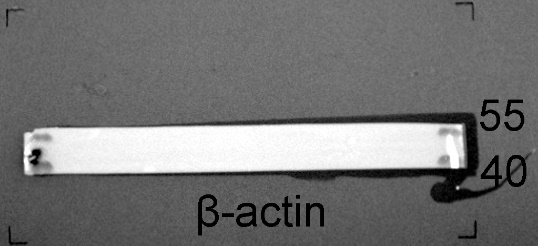

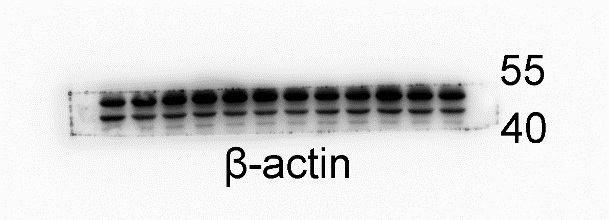

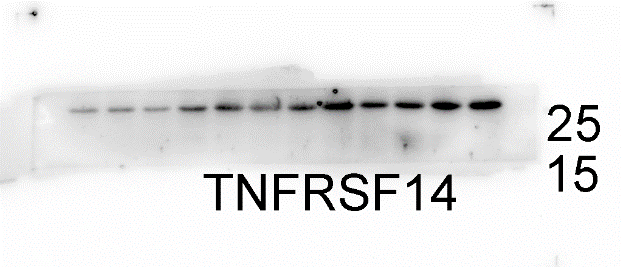


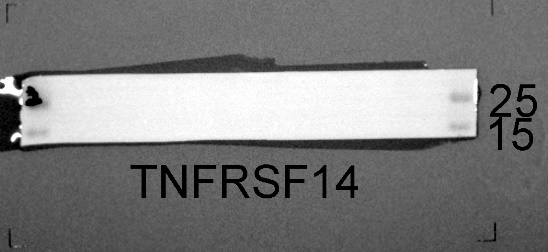


**Source of Fig 1F**


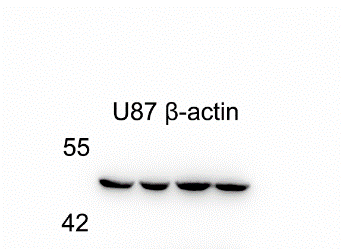

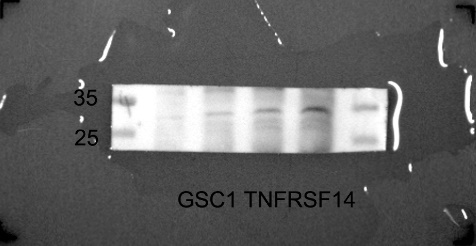

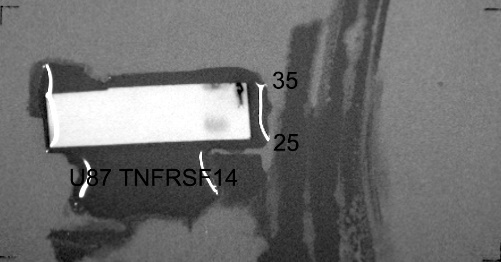

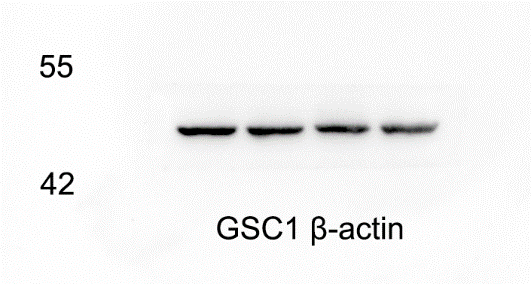

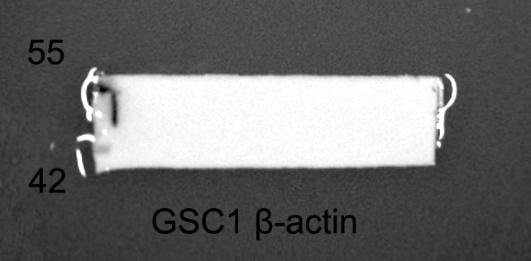

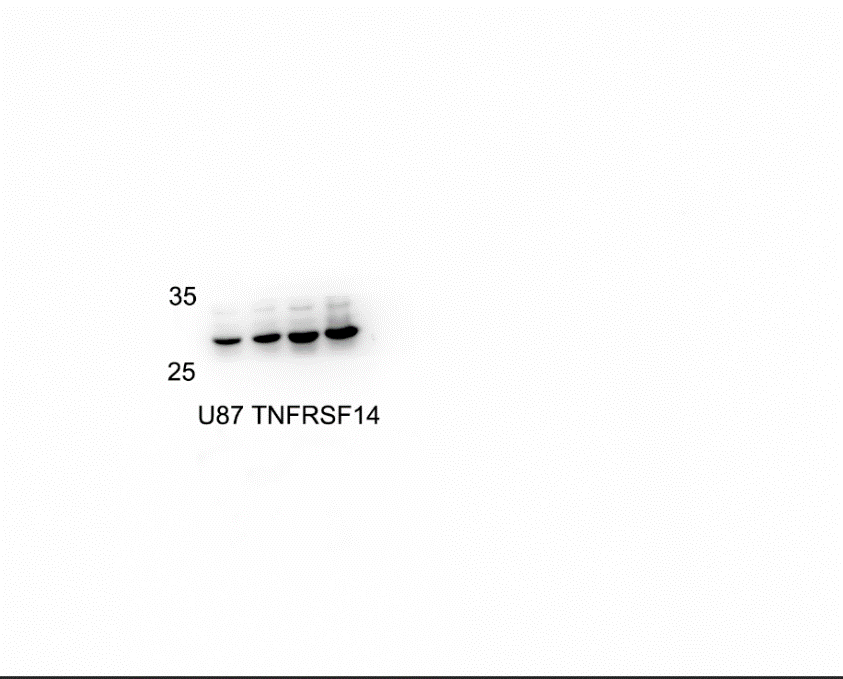


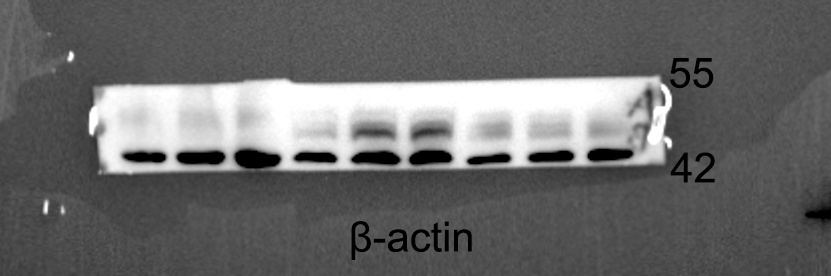
**Source of Fig 1K**


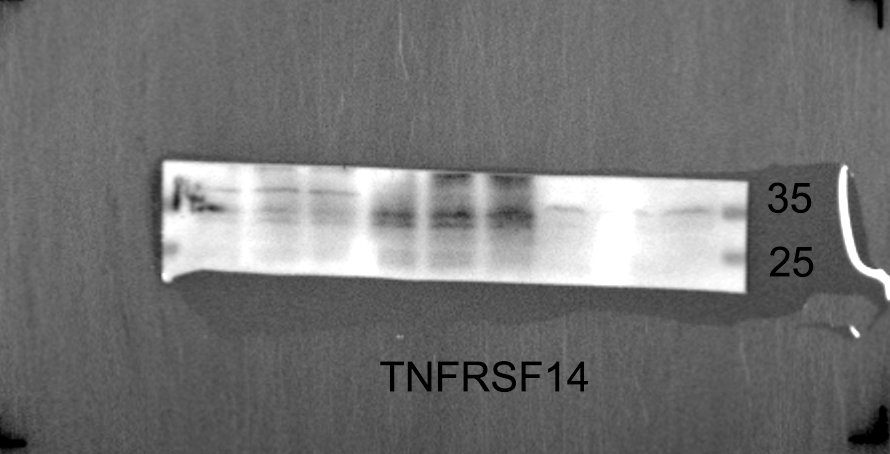


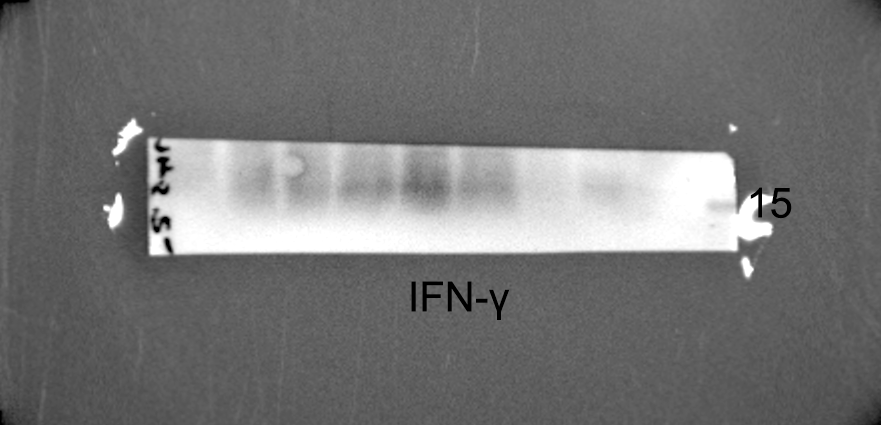


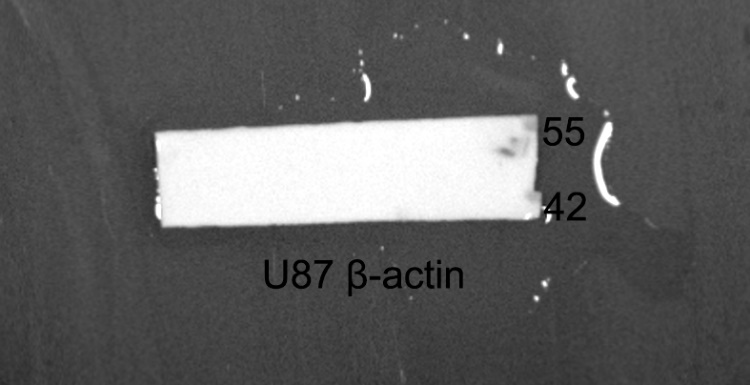

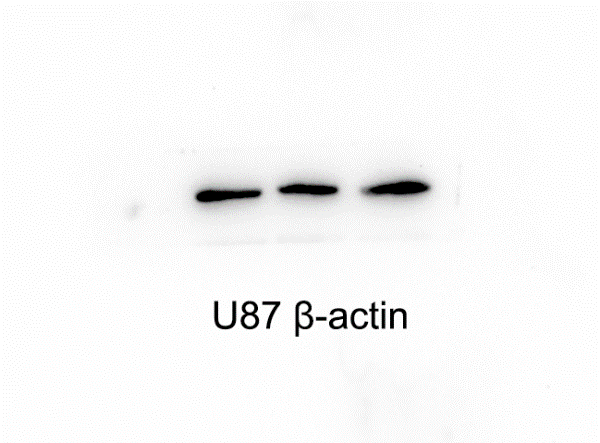

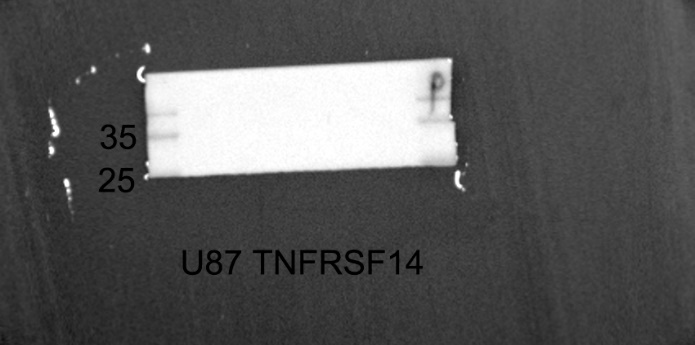

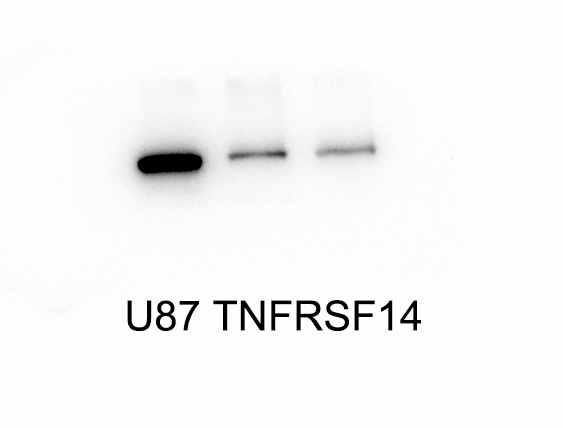

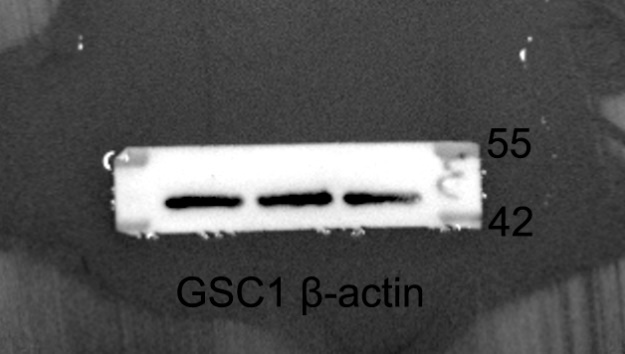

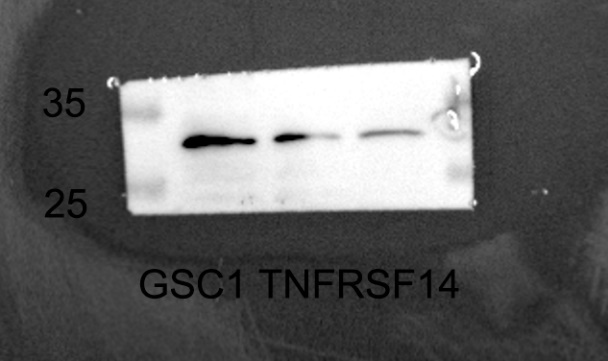
**Source of Fig 2A**


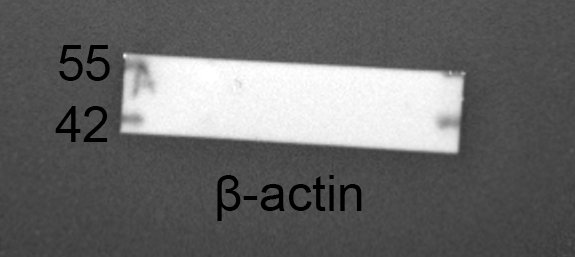

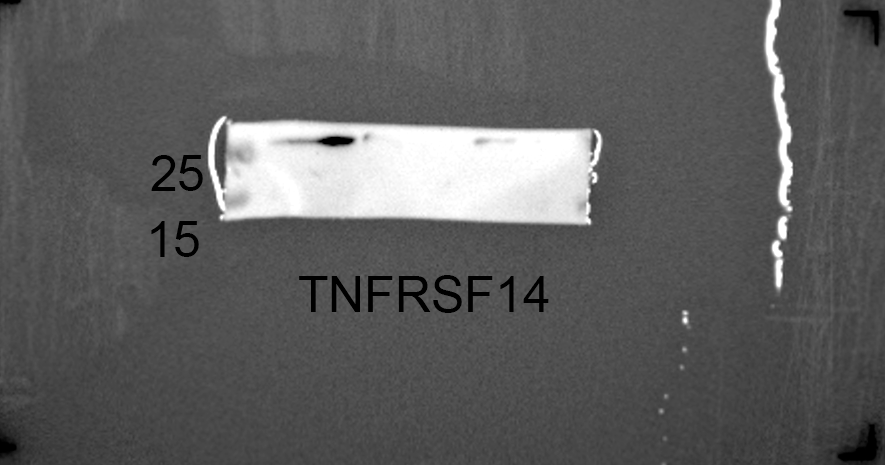
**Source of Fig 2H**


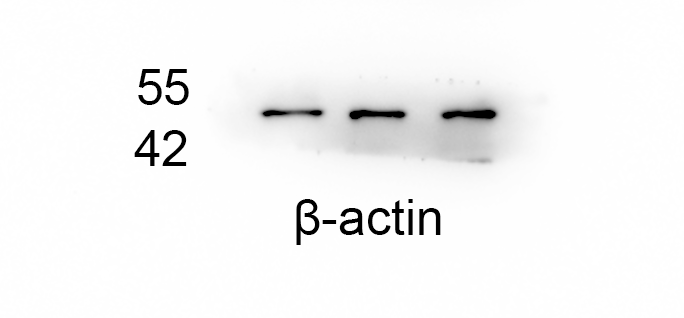


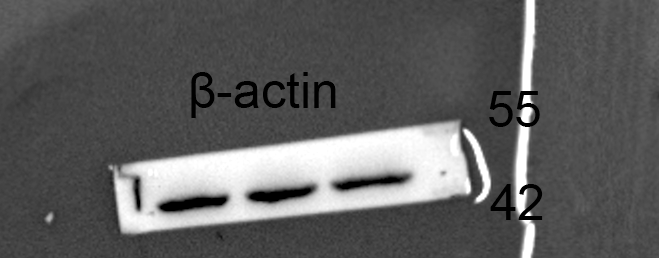

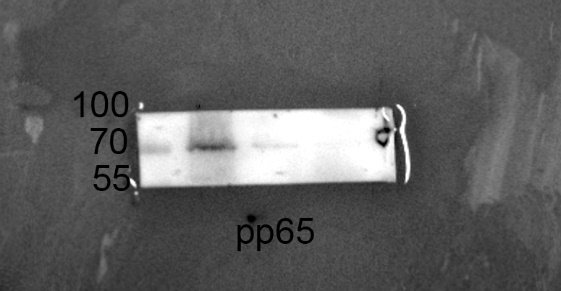

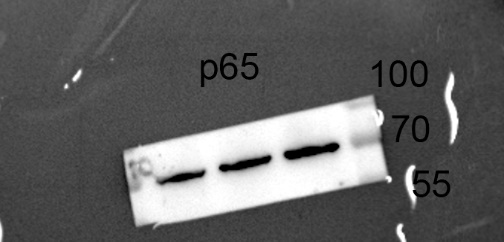

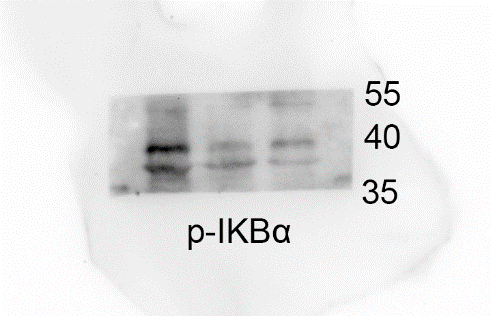

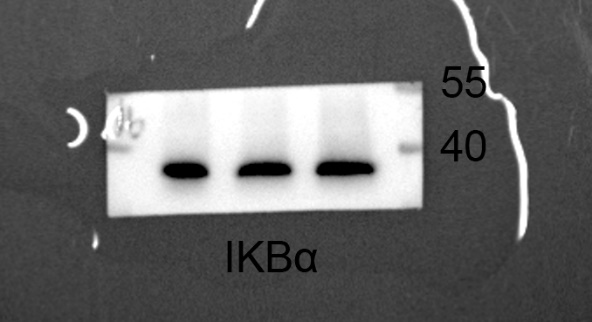

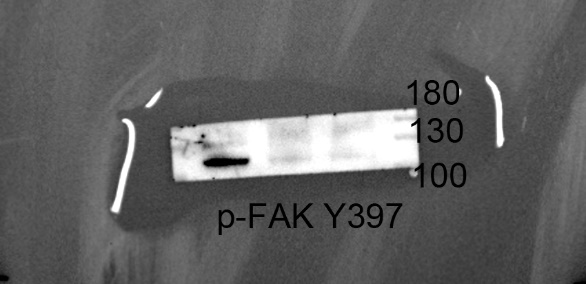

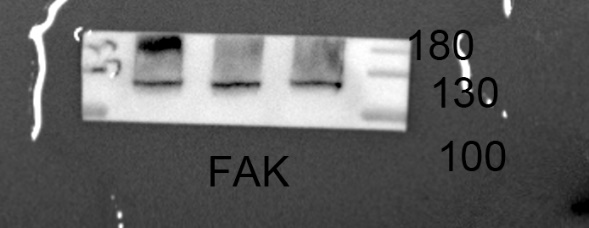

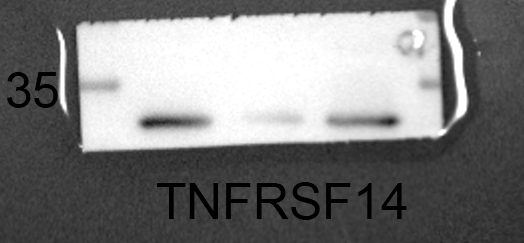
**Source of Fig 4B left panel (GSC1)**

**Source of Fig 4B right panel (U87)**


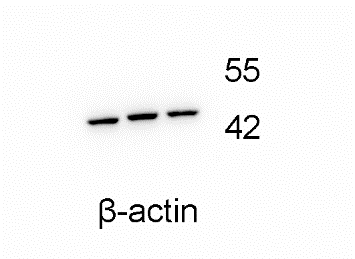

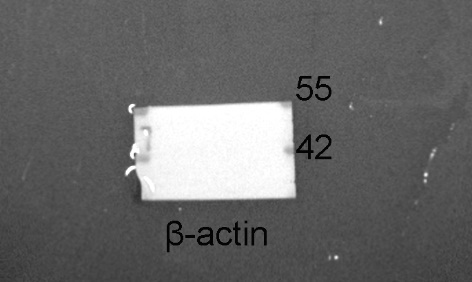

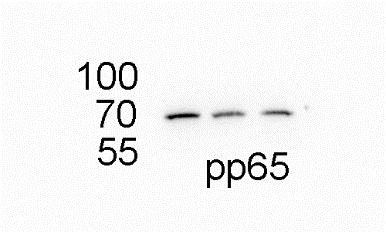

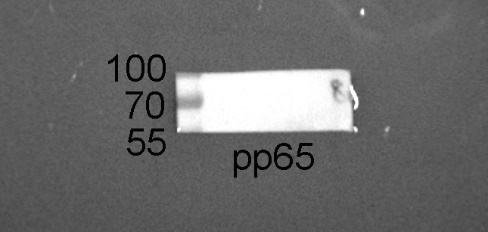

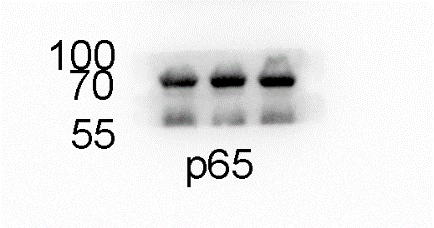

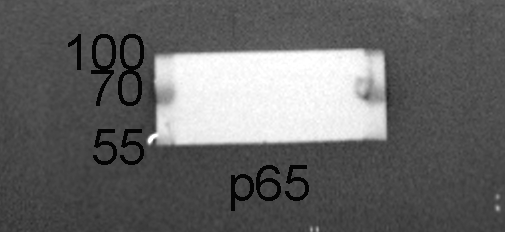

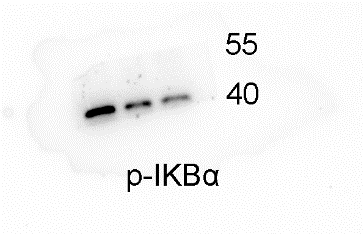

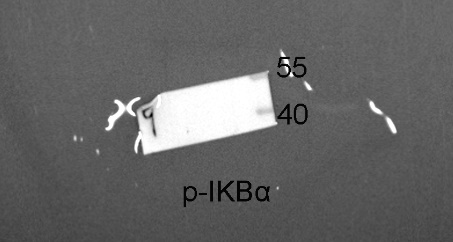

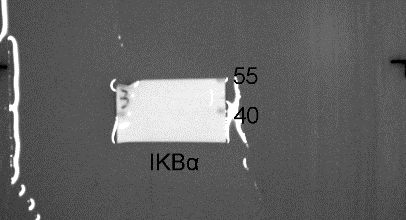

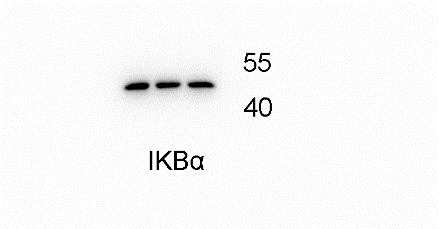

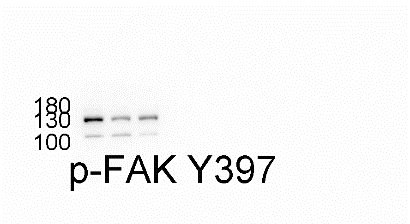

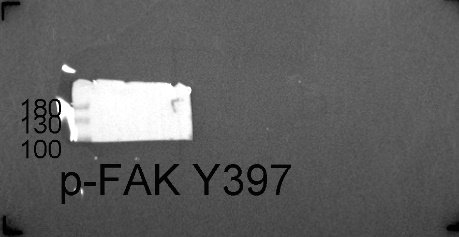

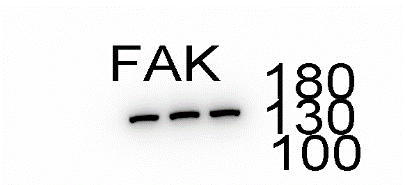

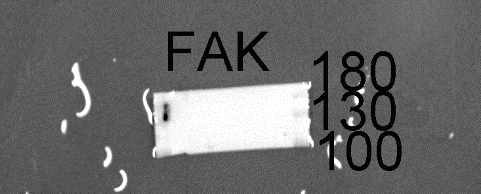

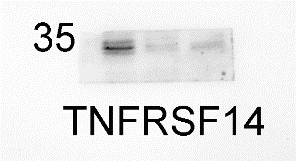

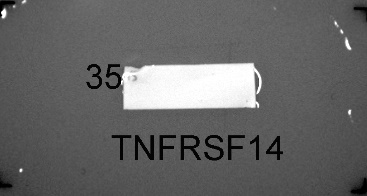


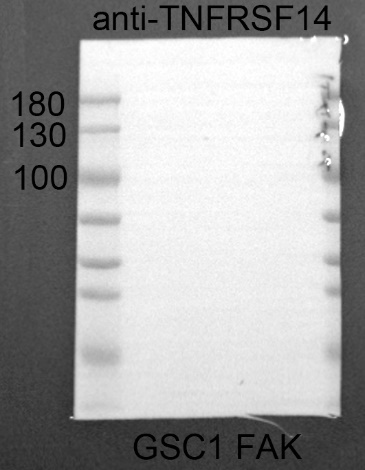

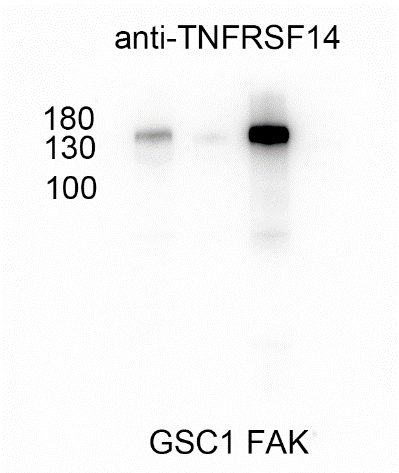

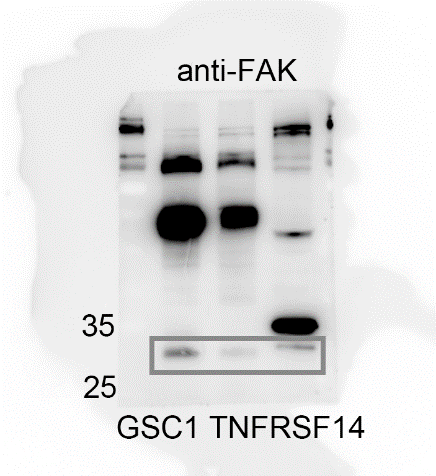

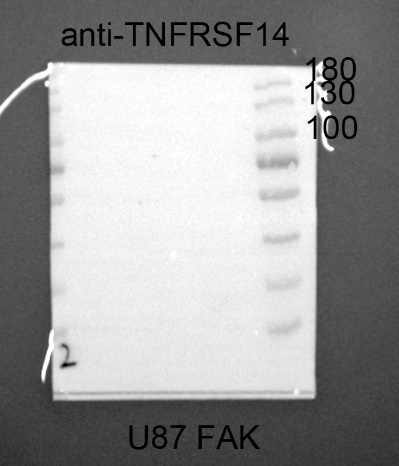

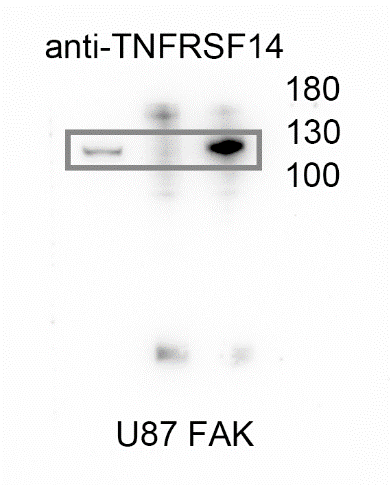

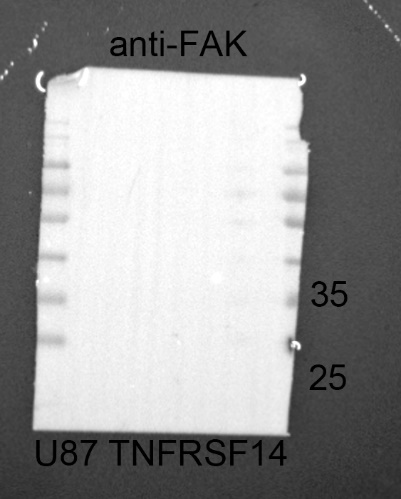

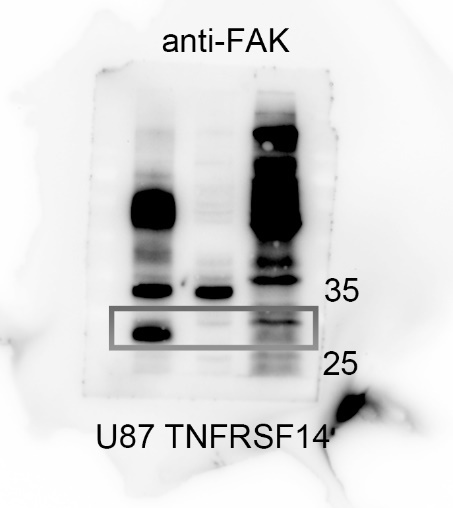
**Source of Fig 4C**


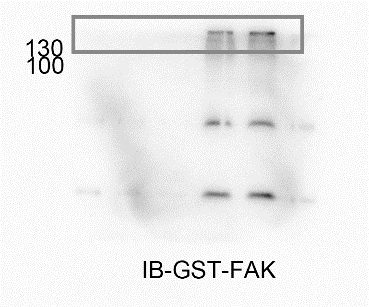

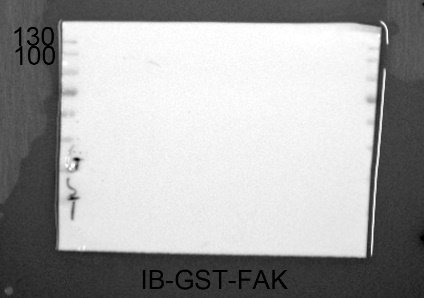

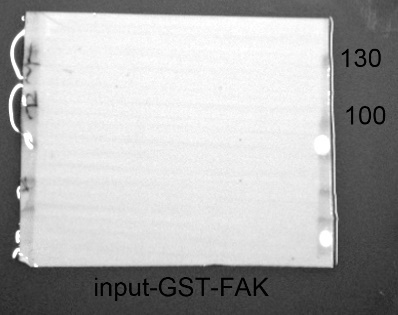

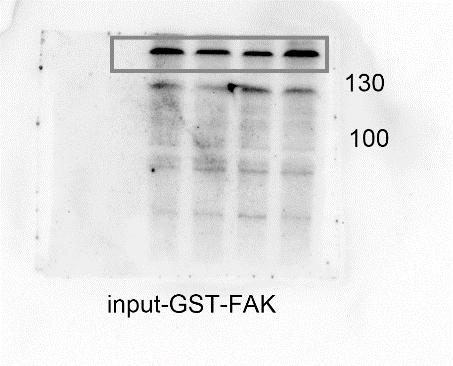

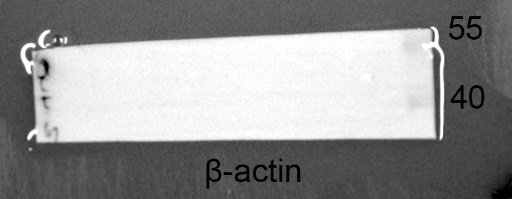

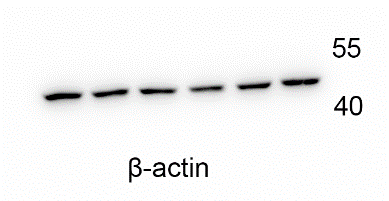

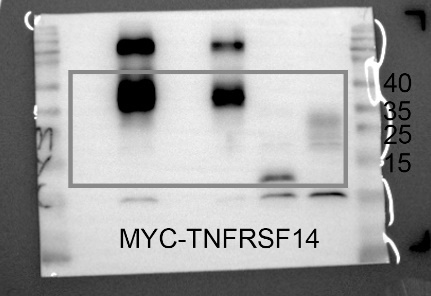
**Source of** **Fig 4D**


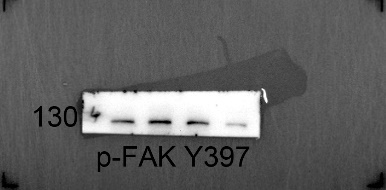

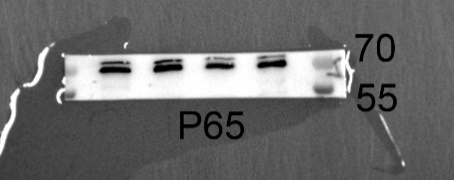

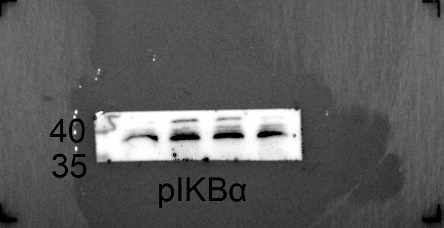

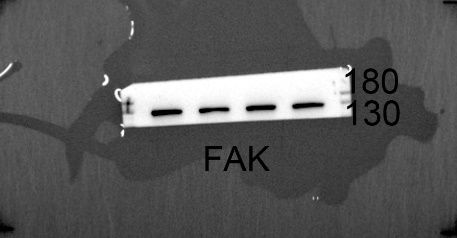

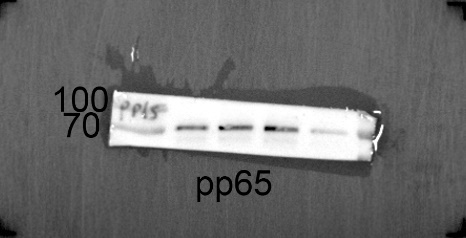

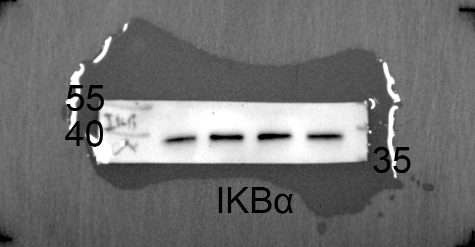

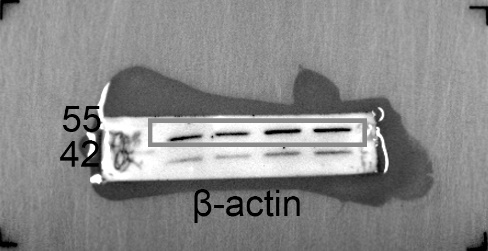
**Source of** **Fig 4E**


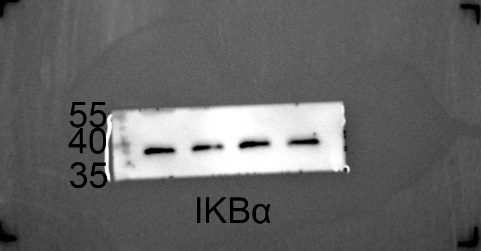

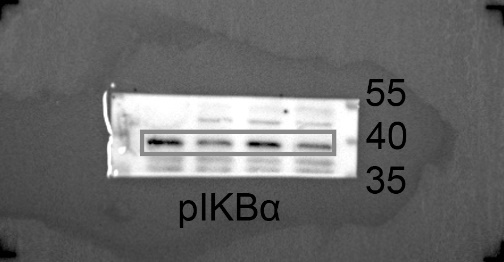

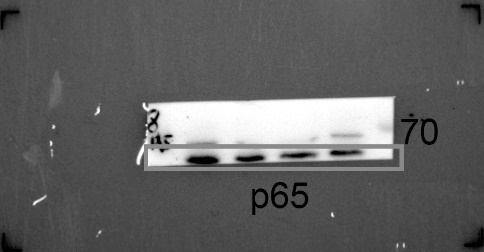

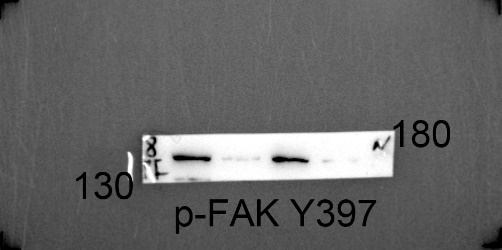

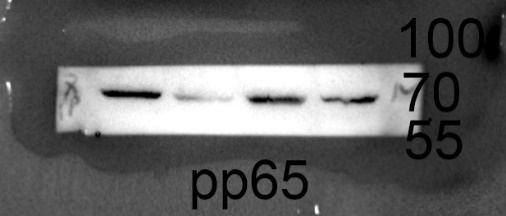

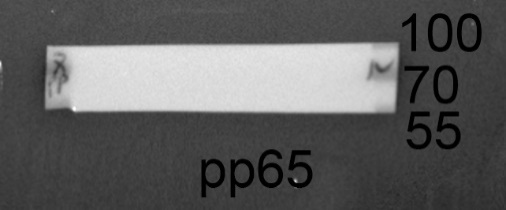

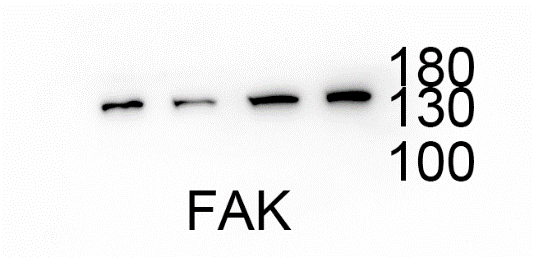

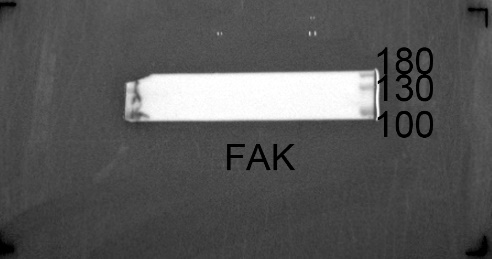

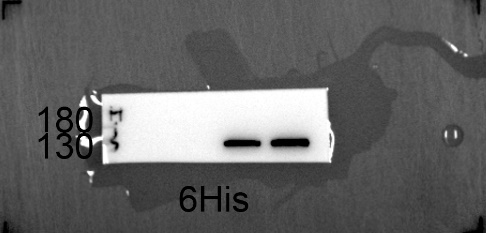
**Source of** **Fig 4F**


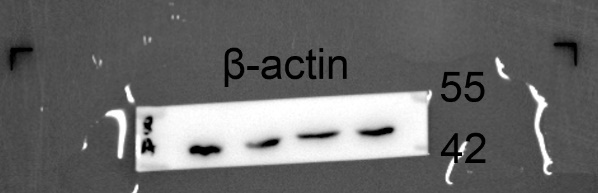

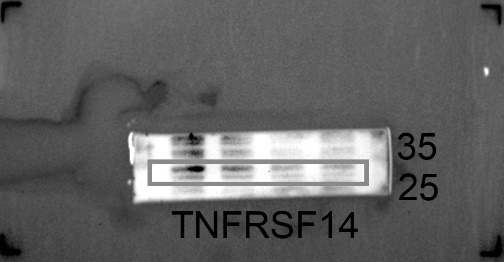


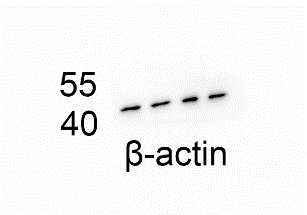

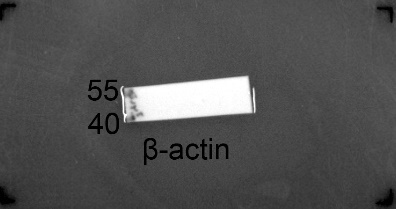

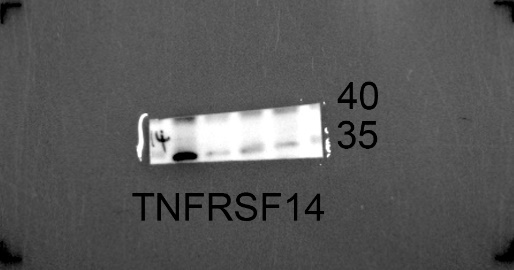

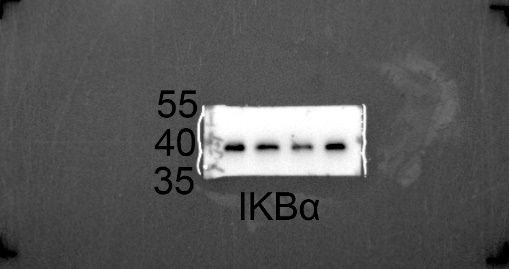

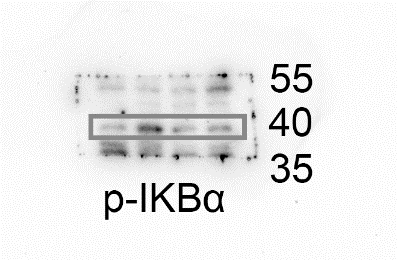

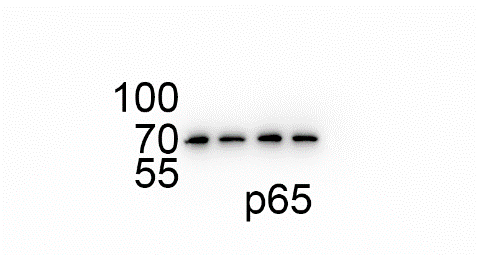

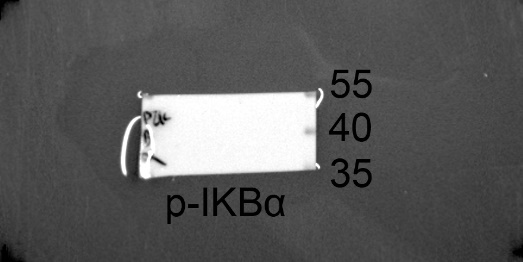

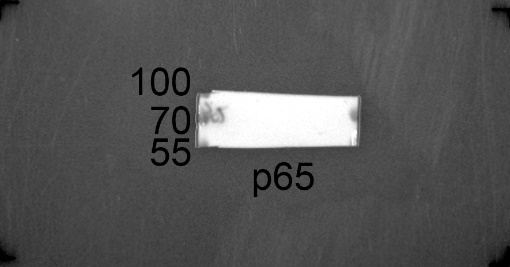

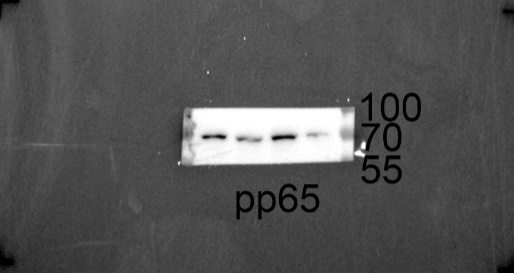

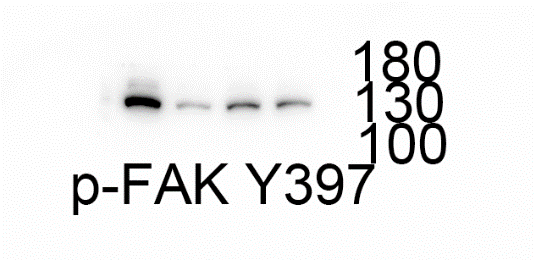

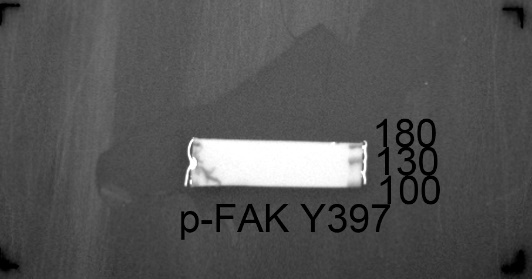

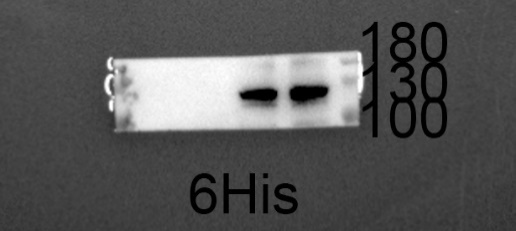

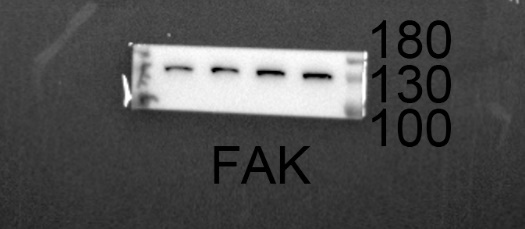
**Source of** **Fig 4G**


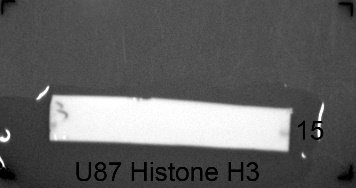

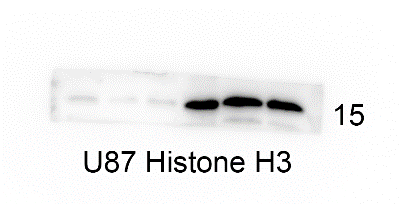

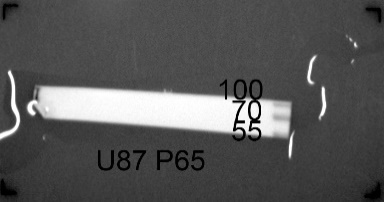

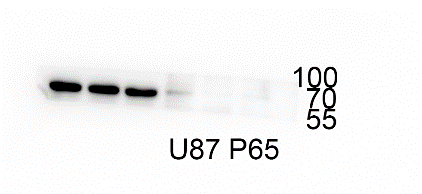

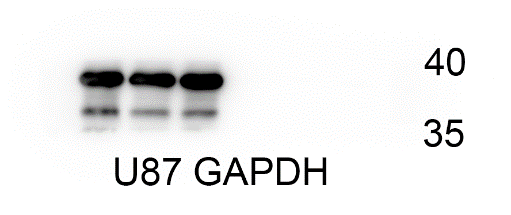

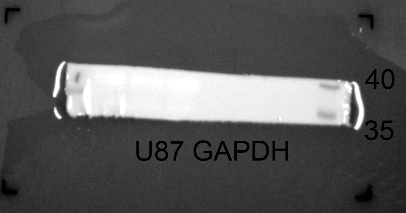

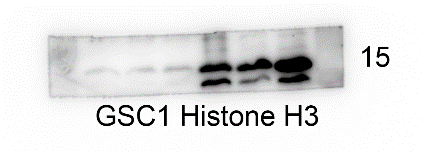

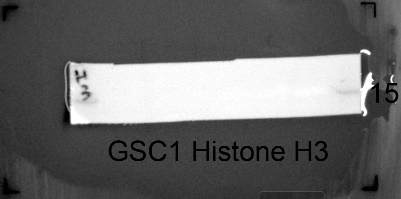

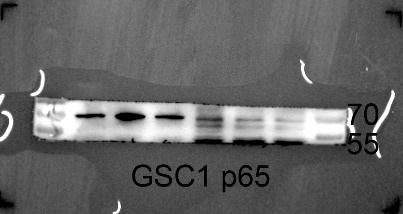
 **Source of** **Fig 4I**

**Source of** **Fig S2C**

**Source of Fig S1I**

TNFRSF14 U87+IFNβ

TNFRSF14 U87+IFNα

β-actin U87+IFNα

β-actin U87+IFNβ

TNFRSF14 GSC1+IFNβ

TNFRSF14 GSC1+IFNα

β-actin GSC1+IFNβ

β-actin GSC1+IFNα

**Source of Fig S2E**

**Source of Fig S4D left (GSC1)**

**Source of Fig S4D right (U87)**

**Source of Fig S4E**

**Source of Fig S4F**

**Source of Fig S4G left (GSC1)**

β-Actin

TNFRSF14

p-p65

p65

p-IKBα

IKBα

p-FAK Y397

FAK

**Source of Fig S4G right (U87)**

β-Actin

TNFRSF14

p-p65

p65

p-IKBα

IKBα

FAK

p-FAK Y397

**Source of Fig S4H** **(GSC1)**

**Source of Fig S4H** **(U87)**

**Figure S1 related to Figure 1. The characterization of TNFRSF14 expression in glioblastoma.**

(A-C) The top 30 immune checkpoints correlated with the score of IFN signaling in GBM datasets (A: CMU n = 208, B: CGGA n = 144, C: TCGA n = 168, Pearson correlation analysis, specific r and p value were showed in Table S6).

(D) Prognosis analysis of CD86, CD48, TIM-3, TNFRSF14 and TNFRSF1B in GBM of CGGA and TCGA (CGGA: n = 69, TCGA: n = 84, log-rank).

(E) Analysis of TNFRSF14 ligands (BTLA and CD160) in single-cell GBM RNA-seq dataset (cell subpopulation distribution map in the left and result of single cell analysis in the right) (GSE131928).

(F) IFN-γ and TNFRSF14 staining analysis of indicated mice brain sections in Fig1L (n = 3, one-way ANOVA).

(G) The expression analysis of IFN-γ and PD-L1 in indicated GBM datasets disclosing that GBM samples with high or low IFN-γ expression didn’t exhibit distinct PD-L1 expression (CGGA: grade II low n =52, high n = 53, grade III low n = 33, high n = 34, grade IV low n = 69, high n = 69; TCGA: grade II low n =111, high n = 112, grade III low n = 122. high n = 123, grade IV low n = 84, high n = 84, t-test).

(H) The combined elevation of IFN-γ and PD-L1 was not correlated with poor survival of GBM (CGGA: low-low n = 42, high-high n = 42, low-high n = 42, high-low n = 42; TCGA: low-low n = 34, high-high n = 35, low-high n = 34, high-low n = 35, log-rank).

(I) Western blotting analysis of TNFRSF14 in indicated GBM cell samples (GSC1 and U87) with indicated IFN-α or IFN-β treatment (0, 20, 50, 100 ng/ml, 48 h).

(n.s. p ≥ 0.05, * p < 0.05, ** p < 0.01, *** p < 0.001, **** p < 0.0001).

**Figure S2 related to Figure 2. TNFRSF14 is involved in regulating migration, invasion, and proliferation of GBM cells.**

(A, B) Representative images of *in vitro* migration (A, representative images related to Fig. 2C) and invasion (B, representative images related to Fig 2D) assays of indicated GBM cells transduced with TNFRSF14 knockdown or control vector, respectively (scale bar, 50 μm).

(C) Representative western blotting images of TNFRSF14 in indicated GBM cells.

(D) MTS assay showing TNFRSF14 overexpression significantly prompted the growth of indicated GBM cells (n = 3, one-way ANOVA).

(E) Representative western blotting images of TNFRSF14 in GL261 cells (relative to shNC).

(F) Survival analysis of mice intracranially transplanted GL261 cells and then with indicated treatment (n = 6, log-rank).

(G) Bioluminescence images of mice brains transplanted with indicated GL261 cells.

(H) H&E and Ki-67 staining images of indicated mice brain section (left scale bar: 500 μm, right scale bar, 25 μm).

(I-K) Analysis of Ki-67 immunohistochemical staining in indicated mice brain sections (n = 3, one-way ANOVA).

(n.s. p ≥ 0.05, * p < 0.05, ** p < 0.01, *** p < 0.001, **** p < 0.0001).

**Figure S3 related to Figure 3. Glioma cell TNFRSF14 promotes the recruitment of TAMs in GBM.**

(A-C) FACS gating strategy of CD206^+^/MHC-II^+^ macrophages (A, related to Fig. 3A upper panel), CD8^+^/IFN-γ^+^ T cells (B, related to Fig 3A middle panel), and CD8^+^ T cells (C, related to Fig 3A lower panel) detection in tumor samples obtained fromGL261 orthotopic xenografting C57BL/6 mice.

(D) Representative images of *in vitro* migration assays of THP1- and BMDM-derived macrophages treated with indicated conditioned medium (scale bar, 50 μm; related to Fig 3E and Fig S3F).

(E) Representative immunohistochemical staining images and analysis (n = 3, one-way ANOVA) of indicated markers in brain section of mice intracranially transplanted with indicated GL261 cells (scale bar, 25 μm).

(F) *In vitro* migration assays of THP1-derived macrophages treated with conditioned medium from indicated GBM cell (relative to shNC, n = 3, one-way ANOVA).

(G) qPCR analyses of indicated markers in THP1-derived macrophages incubated with indicated conditioned medium treatment (relative to shNC, n = 3, one-way ANOVA).

(H) FACS gating strategy of BMDMs.

(I) FACS gating strategy of CD163^+^/CD80^+^ cell detection in THP1-derived macrophages treated with conditioned medium (related to Fig 3G and Fig S3I).

(J) Flow cytometry analysis of CD163 and CD80 in THP1-derived macrophages with indicated conditioned medium treatment (n = 3, one-way ANOVA).

(n.s. p ≥ 0.05, * p < 0.05, ** p < 0.01, *** p < 0.001, **** p < 0.0001).

**Figure S4 related to Figure 4. Defactinib, a FAK inhibitor, efficiently restrains the migration and invasion of GBM cells.**

(A, B) FAK inhibitor, Defactinib, efficiently inhibits enhanced proliferation (A), migration and invasion (B) capabilities of indicated glioma cells inducing by TNFRSF14 overexpression (relative to Vector, n = 3, one-way ANOVA).

(C) Representative images of *in vitro* cell migration (left) and invasion (right) assay of GSC1 and U87 cells with indicated treatment (related to Fig S4B, scale bar, 50 μm).

(D) Representative western blotting images of indicated markers in GSC1 and U87 treated with Defactinib after TNFRSF14 overexpression.

(E) Co-immunoprecipitation analysis of TNFRSF14 and FAK interaction in HEK293 cells.

(F) Western blotting of indicated markers in HEK293 cells transfected with FAK wild type or Y397F mutant vector.

(G) Western blotting analysis of indicated GBM cells transfected with siFAK or control vector, respectively.

(H) Western blotting analysis of nucleus and cytoplasm p65 expression in indicated GBM cells transfected with siFAK or control vector, respectively.

(I) Transfection validation by IF of HEK293 cell transfected with p65 wild type or nuclear localization sequence mutant vector.

(n.s. p ≥ 0.05, * p < 0.05, ** p < 0.01, *** p < 0.001, **** p < 0.0001).

**Figure S5 related to Figure 5. Malignant cell TNFRSF14/FAK regulates macrophage migration through NF-κB mediating CXCL1 and CXCL5 transcription.**

(A) Representative images of *in vitro* migration assay of THP1-derived macrophages with indicated treatment (scale bar, 50 μm; related to Fig 5E).

(B) The schematic graph of mutant plasmids of promoter of CXCL1 and CXCL5.

**Figure S6 related to Figure 6. Anti-TNFRSF14 treatment remodels GBM tumor environment.**

(A, B) Expression analysis of CD206 (A) and ARG1 (B) in macrophage of mouse GBM tissues receiving indicated treatment.

(C-E) Expression analysis of TNF-α (C), CD48 (D) and Ki67 (E) in T cell of mouse glioma tissues receiving indicated treatment.

**Figure S7 related to Figure 6 Anti-TNFRSF14 and anti-PD-L1 treatment remodeled GBM tumor environment.**

(A) Schematic graph of anti-TNFRSF14 and anti-PD-L1 treatment in indicated GBM mice model.

(B) Survival analysis of mice intracranially transplanted GL261 cells and then with indicated treatment (n = 6, log-rank).

(C) Representative images and analysis of H&E (scale bar, 500 μm) and Ki-67 immunohistochemical staining (scale bar, 25 μm) of indicated mice brain section.

(D) Representative bioluminescence brain images of mice intracranially transplanted GL261 cells and then with indicated treatment.

(E) Representative H&E (scale bar, 500 μm) and Ki-67 immunohistochemical staining images (scale bar, 25 μm) of indicated mice brain section.

(F) Analysis of Ki-67 immunohistochemical staining in indicated mice brain sections (n = 3, one-way ANOVA).

(G, H) Representative immunohistochemical staining images (G) and analysis (H) of indicated markers (CD206, CD86, CD8, and perforin) in brain sections from C57BL/6 mice intracranially transplanted with GL261 cells and then received anti-TNFRSF14, anti-PD-L1, or combination treatment indicated in Fig. S7A (scale bar, 25 μm).

(I) Representative immunohistochemical images of TNFRSF14 in indicated GBM mice brain sections of Fig. S6A (n = 3, scale bar, 25 μm).

(J) Representative H&E images of liver and kidney from mice with indicated anti-TNFRSF14, anti-PD-L1, or combination treatment, respectively.

(n.s. p ≥ 0.05, * p < 0.05, ** p < 0.01, *** p < 0.001, **** p < 0.0001).
